# Estimating 24-h urinary sodium/potassium ratio from casual (‘spot’) urinary sodium/potassium ratio: the INTERSALT Study

**DOI:** 10.1093/ije/dyw287

**Published:** 2016-12-30

**Authors:** Toshiyuki Iwahori, Katsuyuki Miura, Hirotsugu Ueshima, Queenie Chan, Alan R Dyer, Paul Elliott, Jeremiah Stamler, for the INTERSALT Research Group

**Affiliations:** 1 Department of Public Health, Shiga University of Medical Science, Shiga, Japan; 2 Research and Development Department, OMRON HEALTHCARE Co., Ltd, Kyoto, Japan; 3 Center for Epidemiologic Research in Asia, Shiga University of Medical Science, Shiga, Japan; 4 Department of Epidemiology and Biostatistics, School of Public Health, Imperial College London, UK; 5 Department of Preventive Medicine, Feinberg School of Medicine, Northwestern University, Chicago, IL, USA

**Keywords:** Na/K ratio, casual urine, 24-h urine, population estimate, individual estimate

## Abstract

**Background:**

Association between casual and 24-h urinary sodium-to-potassium (Na/K) ratio is well recognized, although it has not been validated in diverse demographic groups. Our aim was to assess utility across and within populations of casual urine to estimate 24-h urinary Na/K ratio using data from the INTERSALT Study.

**Methods:**

The INTERSALT Study collected cross-sectional standardized data on casual urinary sodium and potassium and also on timed 24-h urinary sodium and potassium for 10 065 individuals from 52 population samples in 32 countries (1985–87). Pearson correlation coefficients and agreement were computed for Na/K ratio of casual urine against 24-h urinary Na/K ratio both at population and individual levels.

**Results:**

Pearson correlation coefficients relating means of 24-h urine and casual urine Na/K ratio were *r* = 0.96 and *r* = 0.69 in analyses across populations and individuals, respectively. Correlations of casual urine Na/creatinine and K/creatinine ratios with 24-h urinary Na and K excretion, respectively, were lower than correlation of casual and 24-h urinary Na/K ratio in analyses across populations and individuals. The bias estimate with the Bland–Altman method, defined as the difference between Na/K ratio of 24-h urine and casual urine, was approximately 0.4 across both populations and individuals. Spread around, the mean bias was higher for individuals than populations.

**Conclusion:**

With appropriate bias correction, casual urine Na/K ratio may be a useful, low-burden alternative method to 24-h urine for estimation of population urinary Na/K ratio. It may also be applicable for assessment of the urinary Na/K ratio of individuals, with use of repeated measurements to reduce measurement error and increase precision.

**Key Messages**

- Casual urine sodium-to-potassium (Na/K) ratio with appropriate bias correction may be useful to estimate 24-h urinary Na/K ratio across different populations.
- Use of casual urine Na/K ratio may offer a low-cost method to monitor Na and K intakes in the population.
- Casual urine Na/K ratio with appropriate bias correction may be a useful, low-burden alternative method to 24-h urine collection for estimation of urinary Na/K ratio for individuals, with use of repeated casual urine collection to improve accuracy and precision.
- The associations of both casual urinary Na/creatinine and K/creatinine ratios with 24-h urinary Na and K excretion respectively, were weaker than association of the casual and 24-h urinary Na/K ratio across different populations and for individuals.

## Introduction

High dietary sodium (Na) and low dietary potassium (K) intakes are associated with adverse blood pressure (BP) levels and excess risks of cardiovascular diseases (CVD).^1–4^ The gold standard for estimating individual daily sodium intake is 24-h urine collection.^5–7^ Similarly, the amount of K excreted in 24-h urine is correlated with dietary K intake.^6–8^ However, 24-h urine specimens are neither easy nor practical to collect for patients at clinics or individuals at home, especially if repeated samples are required to estimate individual intake.^9^

The Na/K ratio in 24-h urine is cross-sectionally associated with BP in most epidemiological studies.^10–17^ The Na/K ratio has been reported to be a superior metric to either Na or K alone in relation to BP^10^^,^^11^^,^^18^; studies also report associations between Na/K ratio and CVD.^19^ However, these associations are not seen in all studies.^20–23^ Association studies using dietary self-report instruments also report similar findings on Na/K ratio.^24–27^ High urinary Na/K ratio is therefore an indicator for reducing Na intake and increasing K intake^1^^,^^2^; the WHO (World Health Organization) has suggested that achieving guidelines for Na and K intakes would yield an Na/K ratio of approximately 1.00.^28^^,^^29^ However, it is much easier to obtain casual (spot) than 24-h urine specimens.^30^ Recently, we found that Na/K ratio from repeated casual urine specimens is useful for estimating 24-h urinary Na/K ratio in normotensive and hypertensive Japanese individuals.^31^^,^^32^ These findings have not been validated in different demographic groups. The present study was undertaken to assess utility across and within populations of casual (spot) urine specimens to estimate 24-h urinary Na/K ratio using highly standardized data from the International Cooperative Study on Salt, Other Factors, and Blood Pressure (INTERSALT).^14^^,^^33^^,^^34^

## Methods

### Population samples and participants

INTERSALT collected cross-sectional standardized data on casual urinary Na and K concentrations, and also on timed 24-h urinary Na and K excretions for 10 079 men and women aged 20–59 years from 52 population samples in 32 countries.^14^^,^^33^^,^^34^ Data on 14 persons were excluded due to missing data on either Na or K excretion; hence, data for 10 065 participants were analysed here. Field work took place between 1985 and 1987. Each study centre was asked to recruit 200 men and women stratified by age and sex from whole-population groups or from samples selected randomly.^33^^,^^34^ Institutional ethics committee approval was obtained for each collaborating centre, and all participants gave informed consent.

Before starting 24-h urine collection, each participant was asked to empty his or her bladder and provide a casual urine specimen. In order to avoid under- and over-collection, start and end times of the 24-h urine collection were supervised by clinic staff.^33^^,^^34^ Urine collections started during the day and were rejected if the participant reported that ‘more than a few drops’ were missing from the collection, if 24-h urinary volumes were less than 250 ml or if the timing of the collection fell outside the range of 20 to 28-h.^33^^,^^34^ Urinary variables were standardized to 24-h equivalent by dividing by the ratio of the actual duration of urine collection to 24-h. Aliquots of the casual and 24-h urinary specimens were sent frozen to a central laboratory (Leuven, Belgium) for biochemical analysis including Na (mmol) and K (mmol) by emission flame photometry. Technical errors in the laboratory were 1.4% for Na and 1.9% for K.

### Statistical analysis

Pearson correlation coefficients for Na/K ratios were calculated to examine the correlation between values for casual urines and corresponding values for 24-h urine specimens as the gold standard. Agreement between the casual urine Na/K ratio and 24-h urine Na/K ratio was examined using the method of Bland and Altman.^35^ Bland–Altman plots, showing mean of casual and 24-h urinary Na/K ratio values vs the difference between the two values, were used to assess mean difference (bias), the upper and lower limits of agreement (mean difference ± 1.96 × standard deviation of difference) between casual urine and 24-h urine, and the difference between the upper and lower limits of agreement (defined as 95% limit of the difference).

## Results

For the 52 population samples, mean 24-h urinary Na/K ratios (mmol/mmol) ranged from 0.01 (Yanomamo, Brazil) to 7.58 (Tianjin, China) (Supplementary Tables 1 and 2, available as Supplementary data at *IJE* online). Mean Na/K ratio in 10 Asian populations (5.04) was higher than in 33 Western populations (2.98).

**Table 1:** Pearson correlation coefficients relating Na/K ratio, Na and K concentration of casual urine with Na/K ratio, Na and K excretion of 24-h urine

		52 populations					10 065 individuals				
		24-h urine collection					24-h urine collection				
		Na/K ratio	Na excretion	K excretion	Na/Cr ratio	K/Cr ratio	Na/K ratio	Na excretion	K excretion	Na/Cr ratio	K/Cr ratio
Casual urine	Na/K ratio	0.96	0.72	–0.67	0.78	–0.57	0.69	0.42	–0.34	0.45	–0.33
	Na concentration	0.74	0.84	–0.42	0.66	–0.67	0.46	0.49	–0.14	0.36	–0.35
	K concentration	–0.79	–0.68	0.62	–0.68	0.68	–0.43	–0.24	0.33	–0.32	0.31
	Na/Cr ratio	0.62	0.83	–0.19	0.97	–0.22	0.37	0.42	–0.06	0.68	0.01
	K/Cr ratio	–0.71	–0.44	0.68	–0.28	0.97	–0.37	–0.14	0.36	0.02	0.67

Na, sodium; K, potassium; Cr, Creatinine. *P* < 0.001 for all coefficients. Units for Na and K concentrations are mmol/L. Units for Na and K excretions are mmol/24 h.

**Table 2:** Pearson correlation coefficients and agreement of casual urinary Na/K ratio with 24-h urine Na/K ratio across the 52 population samples

		Correlation coefficient between casual and 24-h urine Na/K ratio	Agreement Bland–Altman plot	
Samples	*n*		Bias	95% of difference
Overall	52	0.96	0.39	1.49
Men	52	0.96	0.42	1.57
Women	52	0.95	0.36	1.64
Ages 20–24	52	0.92	0.36	2.34
Ages 25–34	52	0.95	0.36	1.73
Ages 35–44	52	0.93	0.38	1.91
Ages 45–54	52	0.96	0.38	1.42
Ages 55–59	52	0.92	0.54	2.26
Non-remote populations	48	0.94	0.42	1.52
Western populations	33	0.92	0.39	1.03
Asian populations	10	0.88	0.56	2.55

Na, sodium; K, potassium. Bias is mean value of the difference of Na/K ratio between 24-h urine and casual urine as defined in the Bland–Altman method; 95% of difference is width between upper limit and lower limit in the Bland–Altman plot. Non-remote population: samples excluded four remote samples among the 52 population samples with low sodium consumption (Xingu, Yanomamo, Kenya and Papua New Guinea samples).

**Figure 1:**
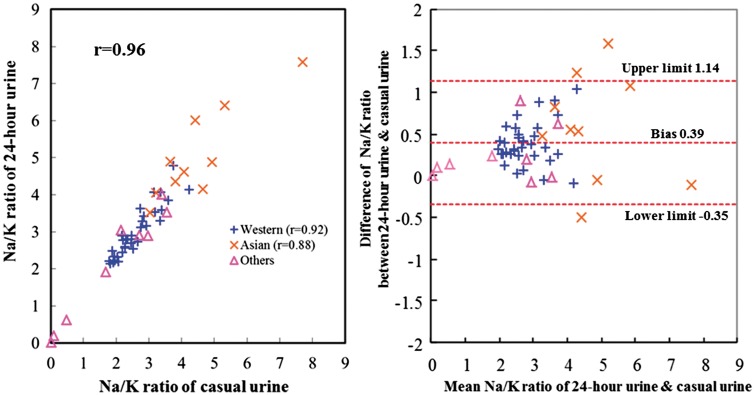
Plot of Na/ K ratio of casual urine vs 24-h urine and Bland–Altman plot (52 population samples). Pearson correlation coefficient between 24-h urinary Na/K ratio and casual urinary Na/K ratio was *r* =* *0.96 across the 52 population samples. Bias between 24-h urinary Na/K ratio and casual urinary Na/K ratio by the Bland–Altman method was 0.39. Others are nine population samples from Argentina, Colombia, Mexico, Trinidad and Tobago, Zimbabwe, Yanomamo and Xingu Indians in Brazil, Kenya and Papua New Guinea; these nine samples were neither Western populations (*n *=33) nor Asian populations (*n *=10).

Casual urinary Na/K ratios were mostly lower than 24-h urine Na/K ratios across samples (Supplementary Tables 1 and 2, available as Supplementary data at *IJE* online). Pearson correlation coefficients for Na/K ratio, Na and K concentrations of casual vs 24-h urine values across populations were highest for Na/K ratio (*r* = 0.96) compared with corresponding *r*-values for casual and 24-h urinary Na or K (Table 1), and were almost identical to those of Na/creatinine (*r* = 0.97) and K/creatinine (*r* = 0.97). Correlations of 24-h urinary Na/K ratio and Na/K ratio of casual urine across samples ranged from *r* =* *0.88 to 0.96 in subgroups categorized by sex, age and across Western/Asian populations (Table 2, Figure 1 and Supplementary Figures 1–3, available as Supplementary data at *IJE* online). After excluding four remote population samples with low Na consumption, correlation coefficients, bias and 95% limit of the difference of the remaining 48 population samples showed similar results compared with all 52 (Table 2).

**Figure 2:**
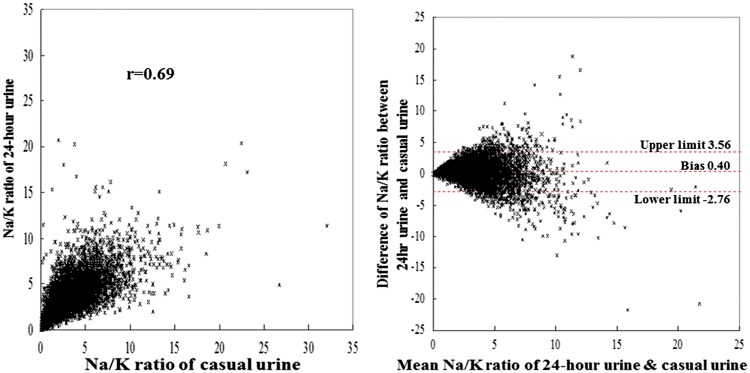
Plot of Na/K ratio of casual urine vs 24-h urine and Bland–Altman plot (10 065 individuals). Pearson correlation coefficient between 24-h urinary Na/K ratio and casual urinary Na/K ratio was *r* =0.69 across 10 065 individuals. The bias between 24-h urinary Na/K ratio and casual urinary Na/K ratio by the Bland–Altman method was 0.40.

For the 10 065 individuals, the overall mean value of the Na/K ratio in 24-h urine was 3.24; Na excretion was 156.0 mmol/24 h; K excretion, 55.2 mmol/24 h; urine volume 1.38 L/24 h (Table 3). Casual urinary Na/K ratios were mostly lower than 24-h urine Na/K ratios across individuals (Table 3). Correlation coefficients for Na/K ratio, Na and K concentrations of casual vs 24-h urine values across individuals were highest for Na/K ratio (*r* =* *0.69) compared with corresponding *r*-values for casual and 24-h urinary Na or K (Table 1), and were almost identical to those of Na/creatinine (*r* = 0.68) and K/creatinine (*r* =* *0.67). Correlations between 24-h urinary Na/K ratio and casual urinary Na/K ratio across individuals ranged from *r* =* *0.47 to 0.81 in subgroups categorized by sex, age, ethnicity and whether or not taking anti-hypertensive medications or K supplements (Table 3, Figure 2 and Supplementary Figures 4–7, available as Supplementary data at *IJE* online). The interaction among subgroups for individual level linear regression analysis was mostly non-significant, although significance was observed in some of the gender, ethnic subgroups which may reflect the large sample size (Supplementary Table 4, available as Supplementary data at *IJE* online).

**Table 3:** Casual and 24-h urinary Na/K ratio, correlation coefficients and agreement in individuals, overall (*n *=10 065) and by demographic stratum

Population sample		Casual urine Na/K ratio		24-h urine Na/K ratio		Correlation coefficients between casual and 24-h urine Na/K ratio	Agreement with Bland–Altman plot	
	*n*	Mean	SD	Mean	SD		Bias	95% of difference
Overall	10 065	2.85	2.15	3.24	1.90	0.69	0.40	6.32
Individual men	5039	2.87	2.11	3.29	1.92	0.70	0.42	6.21
Individual women	5026	2.83	2.18	3.20	1.89	0.68	0.37	6.42
Individuals aged 20–24	1137	2.93	2.39	3.30	2.03	0.72	0.37	6.60
Individuals aged 25–34	2662	2.97	2.21	3.34	1.94	0.68	0.37	6.56
Individuals aged 35–44	2533	2.78	2.09	3.15	1.86	0.69	0.38	6.22
Individuals aged 45–54	2564	2.86	2.08	3.25	1.84	0.69	0.40	6.10
Individuals aged 55–59	1169	2.66	1.97	3.17	1.93	0.68	0.51	6.17
White individuals	5853	2.51	1.56	2.84	1.20	0.58	0.33	5.15
Black individuals	980	2.89	2.10	3.57	2.16	0.47	0.69	8.57
Native American individuals	561	1.10	2.13	1.13	1.79	0.81	0.04	4.91
Asian-Indian individuals	406	3.95	2.47	4.17	1.84	0.70	0.22	6.90
East Asian individuals	1578	4.61	2.79	5.26	2.16	0.64	0.65	8.51
Individuals of other ethnicities (others)	687	2.48	1.93	2.76	1.92	0.70	0.31	5.85
Individuals taking neither anti-hypertensive medications nor potassium	7629	2.92	2.19	3.30	1.94	0.72	0.38	6.16
Individuals taking anti-hypertensive medications or potassium	2436	2.63	1.99	3.08	1.76	0.58	0.45	6.80

Na, sodium; K, potassium; SD, standard deviation. Bias is mean value of the difference of Na/K ratio between 24-h urine collection and casual urine as defined in the Bland–Altman method. 95% of difference is width between upper limit and lower limit in the Bland–Altman plot. Anti-hypertensive medications include potassium sparing diuretics, other diuretics and other anti-hypertensive drugs affecting BP. East Asian individuals are defined as Chinese, Japanese and Korean individuals. Details of the definition of ethnic groups are defined.^33^

The associations of both casual urinary Na/creatinine and K/creatinine ratios with 24-h urinary Na and K excretion, respectively, were weaker compared with association of the casual and 24-h urinary Na/K ratio across the 52 population samples and for the 10 065 individuals (Table 1).

Across the 52 population samples and the 10 065 individuals, the bias estimate with the Bland–Altman method, defined as the difference between Na/K ratio of 24-h urine collection and casual urine, was approximately 0.4 (Tables 2 and 3 and Figures 1 and 2). For subgroups categorized by age, sex and anti-hypertensive medication use, the bias ranged from 0.36 (women, aged 20–24 and aged 25–34) to 0.56 (Asian populations) across samples and 0.04 (Native American) to 0.69 (Black) for individuals (Tables 2 and 3 and Supplementary Figures 1–7, available as Supplementary data at *IJE* online).

Estimates of the 95% limit of the difference with the Bland–Altman method were more than 4-fold larger for the 10 065 individuals than in populations (6.32 vs 1.49) (Tables 2 and 3, Figures 1 and 2 and Supplementary Figures 1–7, available as Supplementary data at *IJE* online).

## Discussion

Main findings from the INTERSALT Study data here are that population mean 24-h urinary Na/K ratio and casual urine Na/K ratio were highly correlated across the 52 population samples allowing first-order correction for systematic differences (bias = 0.4) between casual urine Na/K ratio and 24-h values at a population level. The bias estimate varied by demographic group, being higher for Asian than Western populations, older (aged 50–59 years) than younger (20–29 years) people and for men compared with women. Casual urine Na/K ratio may be a useful, low-burden, low-cost alternative method to 24-h urine collection for estimation of population urinary Na/K ratio level, though calibration vs 24-h urine collection in a subsample is recommended to check validity of the INTERSALT bias estimate in the specific population.

For individuals (*n*= 10 065), although bias was similar to the population-level bias, spread around the mean bias was larger, reflecting diurnal variability and ‘measurement error’ in estimation of the Na/K ratio based on a single casual urine specimen. We suggest that Na/K ratio in casual urine at the individual level may also be a useful proxy for 24-h values if repeated measurements are available to reduce measurement error and increase precision.^31^^,^^32^

The gold standard for estimating individual daily Na intake and K intake is 24-h urine collection.^6–9^ However, obtaining a 24-h urine collection and ensuring its integrity and completeness are major difficulties for public health surveillance and etiological studies that require measurements of Na and K at population or individual level. Error due to under- or over-collection of a 24-h urine specimen should be lower for Na/K ratio compared with Na or K alone, since the ratio is independent of urine volume and is also independent of excretion of creatinine, which may degrade if specimens are not well temperature-controlled.^33^^,^^36–38^ This reduced error might explain the higher correlations seen for Na/K ratio than for Na or K when comparing casual urine and 24-h urine values. Methods for estimating 24-h urine Na excretion from casual urine specimens have been proposed at a population level,^39–41^ but the performance of these methods varies according to time of casual urine collection and the population under study,^42^ and therefore population-specific calibration against 24-h values has been proposed.^39^

Casual urinary Na/K ratio usually fluctuates during the day, and may reflect the balance of ingestion, hydration and sweating.^43–45^ Recent dietary intake is reflected in casual urine Na/K ratio, such that there is increased value after ingesting foods with high Na content and decreased value when such foods are avoided. Large circadian and day-to-day variability in casual urinary Na and K concentrations, urine volume and voiding frequency may reduce ability to estimate 24-h excretion of Na and K alone from casual urine specimens, without use of demographic, anthropometric and additional urinary variables in the estimation procedures. Brown *et al.* reported individual estimates for 24-h Na excretion from casual urine specimens using the INTERSALT data.^39^ In their report, they used multiple variables in the estimating procedures for Na such as urinary creatinine and K, body mass index, age and region.^39^ The correlation of estimated 24-h Na excretion from casual urine with observed 24-h urine Na excretion was 0.79, and bias estimates were small, but trends were observed in Bland–Altman plots showing larger estimated error with higher Na excretion.^39^ In the present study, we did not use any variables in the estimation procedures other than urinary Na/K ratio itself, as the Na/K ratio is not dependent on urinary volume and may also correct to some extent for body size. This makes estimation of the Na/K ratio from casual urines more straightforward than for Na or K alone.

In the INTERSALT Study, casual urine was collected in the daytime; our findings indicate a lower daytime casual urinary Na/K ratio than the mean Na/K ratio throughout the 24 h. This is consistent with results of our previous study in Japanese normotensive and hypertensive participants, where Na/K ratio in randomly selected daytime casual urine was lower than the mean casual urinary Na/K ratio throughout the 24 h.^31^^,^^32^ Studies in Western populations also showed lower Na/K ratio in the daytime than in 24 h,^46^^,^^47^ and lower Na/K ratio was observed in the morning and afternoon than in the evening and overnight.^48^ Thus, casual urine Na/K ratio with appropriate bias correction may be useful for estimation of 24-h urinary Na/K ratio across different populations. This circadian fluctuation in Na/K ratio may be due either to hormonal diurnal rhythms or postprandial surges, though previous studies do not suggest that food intake is an important contributor to the circadian rhythm.^49–51^

A limitation of the present study is that the casual and 24-h urinary data were collected only once in the majority of individuals, limiting the ability to correct for measurement error due to high day-to-day variability in urinary Na and K excretion. With more specimens of casual urine and further repeated 24-h urine collections, stronger associations may be observed compared with the data presented here. Thus, it is reasonable to infer that the data presented here provide an underestimate of correlations and levels of agreement compared with the repeated collection of both casual urine Na/K ratio and 24-h urine Na/K ratio. Repeated casual urine Na/K ratio increases precision of the individual level measurement of Na/K ratio and may result in better estimates in relation to risk of cardiovascular events compared with use of single casual urinary Na and K in future association studies.^31^^,^^32^^,^^52^ The INTERSALT data were derived from general population samples at ages 20–59 years. It is unknown whether our findings are applicable to individuals outside these age ranges or to patients with various diseases, e.g. diabetes, chronic kidney disease and atherosclerotic diseases.

Anti-hypertensive medications used during the 1980s when the INTERSALT data were collected were mainly diuretics; hence, an assumption of our analyses is that diuretics do not affect the association between casual urinary Na/K ratio and 24-h urinary Na/K ratio. In our previous study, the correlations and agreement between mean values of 4–7 repeated randomly selected casual urinary Na/K ratios and 7-day 24-h urinary Na/K ratios in Japanese hypertensive individuals with and without anti-hypertensive medications showed similar results.^32^ In this previous study, participants taking anti-hypertensive medication were mainly using calcium channel blockers, Angiotensin 2 receptor blockers (ARBs) and the combination of these two.^32^ Therefore, it appears that our findings are robust to class of use of anti-hypertensive medication.

Most association studies of Na/K ratio and BP are cross-sectional and the lack of follow-up is a limitation of these studies. However, the WHO has suggested that achieving its guidelines for both Na and K intakes would yield an Na/K ratio of approximately 1.00.^28^^,^^29^ However, there are any number of combinations of Na and K that could produce a Na/K ratio of 1.00, e.g. both larger Na and K, both intermediate Na and K, both smaller Na and K. Experimental studies decades ago demonstrated that rats ingesting the same level of Na/K ratio with larger Na and K intakes developed higher BP compared with smaller Na and K intakes.^53^ Thus, there might be some difference with respect to BP among the various possible combinations of Na and K even with the same level of Na/K ratio in humans; however, this has not yet been evaluated. Further investigation is needed to address this issue.

In conclusion, our findings suggest that casual urine Na/K ratio with appropriate bias correction for systematic difference, e.g. 0.4, may be a useful, low-burden alternative method to 24-h urine collection for estimation of urinary Na/K ratio across different populations. This may also be the case for individuals with repeated casual urine collection to improve accuracy and precision.

## Supplementary Data

Supplementary data are available at *IJE* online.

## Funding

The International Cooperative Study on Salt, Other Factors, and Blood Pressure (INTERSALT) was supported by the Council on Epidemiology and Prevention of the World Heart Federation (Geneva, Switzerland); the World Health Organization (Geneva, Switzerland); the International Society of Hypertension (Ware, UK); the Wellcome Trust (London, UK); the National Heart, Lung, and Blood Institute, National Institutes of Health (Bethesda, MD, USA); the Heart and Stroke Foundation of Canada (Ottawa, Ontario, Canada); the British Heart Foundation (London, UK); the Japan Heart Foundation (Tokyo, Japan); Netherlands Heart Foundation (Den Haag, The Netherlands); the Chicago Health Research Foundation (Chicago, IL, USA); the Belgian National Research Foundation (Brussels, Belgium); Parastatal Insurance Company (Brussels, Belgium); and by many national agencies supporting local studies. Dr Paul Elliott is supported by the National Institute for Health Research (NIHR) Imperial College Healthcare NHS Trust (ICHNT) and Imperial College Biomedical Research Centre (BRC) (grant number P38084), the MRC-PHE Centre for Environment and Health, and the NIHR Health Protection Research Unit on Health Impact of Environmental Hazards; he is an NIHR Senior Investigator.

## Supplementary Material

Supplementary Data
