# Supplementary material for: Estimating 24-h urinary sodium/potassium ratio from casual (‘spot’) urinary sodium/potassium ratio: the INTERSALT Study
Source: Int J Epidemiol. 2016 Dec 30;46(5):1564–72. doi: 10.1093/ije/dyw287 (PMC5837629; doi:10.1093/ije/dyw287)
Supplement: Supplementary Data [file dyw287_supplemental_final_wo_highlight.doc]

**Supplementary Table 1. Characteristics of study participants by population**

| **Population Sample** | |  | 24-hour urine sodium, mmol/24h | | 24-hour urine potassium, mmol/24h | | 24-hour urine Na/K ratio, mmol/mmol | | Casual urine Na/K ratio, mmol/mmol | | Urine volume, L/24h | |
| --- | --- | --- | --- | --- | --- | --- | --- | --- | --- | --- | --- | --- |
|  |  | N | Mean | SD | Mean | SD | Mean | SD | Mean | SD | Mean | SD |
| Buenos Aires | Argentina | 200 | 155.8 | 61.2 | 56.5 | 17.9 | 2.91 | 1.20 | 2.70 | 1.65 | 1.43 | 0.62 |
| Charleroi | Belgium | 157 | 142.4 | 55.0 | 63.9 | 20.2 | 2.34 | 0.89 | 1.94 | 1.20 | 1.58 | 0.75 |
| Ghent | Belgium | 200 | 147.7 | 58.8 | 70.0 | 24.4 | 2.20 | 0.75 | 2.07 | 1.18 | 1.58 | 0.68 |
| Xingu | Brazil | 198 | 12.3 | 21.5 | 87.0 | 44.2 | 0.20 | 0.47 | 0.09 | 0.21 | 1.58 | 0.79 |
| Yanomamo | Brazil | 195 | 0.9 | 2.5 | 63.4 | 32.1 | 0.01 | 0.04 | 0.01 | 0.02 | 1.08 | 0.57 |
| Labrador | Canada | 161 | 153.4 | 84.3 | 46.2 | 20.2 | 3.62 | 2.05 | 2.74 | 1.92 | 2.00 | 0.90 |
| St John's | Canada | 200 | 200.1 | 84.7 | 59.7 | 23.1 | 3.59 | 1.50 | 3.40 | 2.32 | 1.73 | 0.61 |
| Tuquerres | Colombia | 191 | 201.8 | 79.3 | 71.7 | 24.2 | 2.90 | 0.93 | 2.97 | 1.40 | 1.72 | 0.51 |
| Glostrup | Denmark | 199 | 140.1 | 55.3 | 66.7 | 22.1 | 2.21 | 0.87 | 1.78 | 1.12 | 1.66 | 0.71 |
| Bernried | Germany (West Germany) | 197 | 167.1 | 64.2 | 71.6 | 22.2 | 2.45 | 0.96 | 2.20 | 1.17 | 1.53 | 0.64 |
| Heidelberg | Germany (West Germany) | 196 | 172.7 | 63.1 | 72.9 | 23.9 | 2.48 | 0.90 | 1.88 | 0.90 | 1.67 | 0.72 |
| Joensuu | Finland | 200 | 170.4 | 62.8 | 76.6 | 24.0 | 2.32 | 0.82 | 2.04 | 1.15 | 1.52 | 0.57 |
| Turku | Finland | 200 | 154.8 | 62.7 | 76.1 | 24.1 | 2.14 | 0.92 | 1.82 | 0.96 | 1.50 | 0.57 |
| Cottbus | Germany (East Germany) | 198 | 147.7 | 64.3 | 55.0 | 18.8 | 2.73 | 0.92 | 2.67 | 1.34 | 1.13 | 0.59 |
| Porcsalma | Hungary | 200 | 198.3 | 84.4 | 49.9 | 18.8 | 4.14 | 1.49 | 4.24 | 2.34 | 1.43 | 0.64 |
| Reykjavik | Iceland | 200 | 138.3 | 48.2 | 65.0 | 21.2 | 2.22 | 0.77 | 1.96 | 1.04 | 1.43 | 0.60 |
| Ladakh | India | 200 | 203.7 | 74.8 | 47.0 | 21.5 | 4.88 | 2.09 | 4.93 | 2.75 | 1.79 | 0.65 |
| New Delhi | India | 199 | 160.7 | 61.1 | 47.4 | 15.3 | 3.52 | 1.17 | 3.04 | 1.70 | 1.39 | 0.72 |
| Bassiano | Italy | 199 | 184.8 | 65.0 | 58.1 | 18.8 | 3.30 | 0.96 | 3.35 | 1.70 | 1.13 | 0.38 |
| Gubbio | Italy | 199 | 175.3 | 64.0 | 55.8 | 17.3 | 3.29 | 1.14 | 2.80 | 1.43 | 1.14 | 0.41 |
| Mirano | Italy | 200 | 174.1 | 61.6 | 57.4 | 15.8 | 3.12 | 1.04 | 2.75 | 2.18 | 1.26 | 0.53 |
| Naples | Italy | 200 | 167.7 | 52.6 | 60.2 | 17.0 | 2.90 | 0.93 | 2.18 | 0.91 | 1.02 | 0.34 |
| Osaka | Japan | 197 | 168.4 | 54.1 | 43.1 | 13.3 | 4.05 | 1.22 | 3.22 | 1.44 | 1.20 | 0.48 |
| Tochigi | Japan | 194 | 180.4 | 70.3 | 46.0 | 18.7 | 4.14 | 1.31 | 4.65 | 2.45 | 1.13 | 0.45 |
| Toyama | Japan | 200 | 212.4 | 64.1 | 48.4 | 15.3 | 4.61 | 1.39 | 4.08 | 1.81 | 1.39 | 0.44 |
| Rambugu-Ndori | Kenya | 176 | 56.9 | 33.0 | 33.4 | 18.3 | 1.92 | 1.05 | 1.68 | 1.33 | 1.08 | 0.51 |
| Dingli | Malta | 200 | 169.8 | 59.7 | 73.6 | 29.1 | 2.57 | 1.18 | 2.27 | 1.56 | 1.56 | 0.75 |
| Tarahumara | Mexico | 172 | 144.7 | 84.3 | 43.5 | 23.9 | 3.53 | 1.54 | 3.54 | 2.57 | 1.54 | 0.55 |
| Zutphen | The Netherlands | 199 | 150.7 | 57.3 | 72.5 | 21.5 | 2.17 | 0.84 | 1.92 | 1.14 | 1.51 | 0.59 |
| Asaro | Papua New Guinea | 162 | 37.7 | 31.7 | 71.4 | 37.2 | 0.63 | 0.57 | 0.48 | 0.52 | 0.67 | 0.34 |
| Beijing | China | 200 | 204.1 | 69.5 | 35.3 | 10.7 | 6.01 | 1.97 | 4.42 | 2.22 | 1.37 | 0.53 |
| Nanning | China | 200 | 169.2 | 62.0 | 27.2 | 8.5 | 6.41 | 2.14 | 5.33 | 2.67 | 1.22 | 0.47 |
| Tianjin | China | 200 | 245.6 | 85.9 | 33.6 | 10.5 | 7.58 | 2.42 | 7.69 | 4.16 | 1.70 | 0.60 |
| Krakow | Poland | 200 | 197.7 | 79.6 | 52.5 | 18.1 | 3.85 | 1.23 | 3.60 | 1.43 | 1.24 | 0.47 |
| Warsaw | Poland | 200 | 181.3 | 80.5 | 46.6 | 18.0 | 4.07 | 1.61 | 3.16 | 1.63 | 1.18 | 0.44 |
| Cartaxo | Portugal | 198 | 182.0 | 76.4 | 65.8 | 25.0 | 2.90 | 1.07 | 2.47 | 1.39 | 1.19 | 0.43 |
| Pusan | South Korea | 198 | 208.3 | 76.3 | 49.3 | 15.8 | 4.36 | 1.42 | 3.81 | 1.87 | 1.49 | 0.52 |
| Moscow | Russia (Soviet Union) | 194 | 161.7 | 62.3 | 48.9 | 16.0 | 3.42 | 1.20 | 2.84 | 1.40 | 1.02 | 0.32 |
| Manresa | Spain | 200 | 174.6 | 67.5 | 67.4 | 20.6 | 2.69 | 0.92 | 2.45 | 1.51 | 1.16 | 0.45 |
| Torrejon | Spain | 200 | 183.2 | 69.0 | 67.1 | 18.4 | 2.80 | 0.96 | 2.47 | 1.29 | 1.31 | 0.43 |
| San Chilo | Taiwan | 181 | 141.5 | 60.1 | 31.7 | 15.0 | 4.89 | 2.13 | 3.65 | 2.04 | 1.16 | 0.49 |
| Plymouth-Bethesda | Trinidad and Tobago | 176 | 116.7 | 57.6 | 40.7 | 17.1 | 3.06 | 1.34 | 2.15 | 1.30 | 0.95 | 0.46 |
| Belfast | United Kingdom | 199 | 150.7 | 56.9 | 56.9 | 18.7 | 2.79 | 1.04 | 2.30 | 1.28 | 1.53 | 0.82 |
| Birmingham | United Kingdom | 200 | 153.1 | 48.0 | 63.0 | 20.4 | 2.59 | 0.98 | 2.27 | 1.24 | 1.74 | 0.65 |
| South Wales | United Kingdom | 199 | 152.5 | 60.8 | 63.1 | 24.4 | 2.54 | 0.86 | 2.51 | 1.50 | 1.55 | 0.74 |
| Chicago | United States | 196 | 140.0 | 66.5 | 53.4 | 21.8 | 2.81 | 1.28 | 2.35 | 1.56 | 1.45 | 0.71 |
| Goodman – Black | United States | 186 | 103.7 | 53.8 | 24.6 | 12.1 | 4.79 | 2.69 | 3.74 | 2.52 | 0.86 | 0.49 |
| Goodman – White | United States | 198 | 130.8 | 57.2 | 45.2 | 21.9 | 3.16 | 1.23 | 2.92 | 1.81 | 1.24 | 0.56 |
| Hawaii | United States | 187 | 143.9 | 77.4 | 44.5 | 23.1 | 3.53 | 1.84 | 3.19 | 1.90 | 1.39 | 0.87 |
| Jackson – Black | United States | 184 | 146.8 | 80.7 | 39.3 | 19.1 | 4.07 | 2.13 | 3.35 | 1.99 | 1.19 | 0.69 |
| Jackson – White | United States | 199 | 141.5 | 57.1 | 56.5 | 22.8 | 2.78 | 1.35 | 2.21 | 1.33 | 1.63 | 0.83 |
| Harare | Zimbabwe | 195 | 140.4 | 62.5 | 37.5 | 13.8 | 4.01 | 2.05 | 3.39 | 2.17 | 1.75 | 0.81 |

Na, sodium; K, potassium; SD, standard deviation.

1. Data for sodium, potassium, Na/K ratio and urine volume are previously published by gender and age in The INTERSALT Co-operative Research Group. Appendix tables. Centre-specific results by age and sex. *J Hum Hypertens*. 1989 Oct;3(5):331-407.

**Supplementary Table 2. Sex-specific Na/K ratio (mmol/mmol) of Spot Urine and 24-hour Urine of population samples**

| Population Sample | | Casual urine | | | | | | 24-hour urine | | | | | |
| --- | --- | --- | --- | --- | --- | --- | --- | --- | --- | --- | --- | --- | --- |
|  |  | Men | | | Women | | | Men | | | Women | | |
|  |  | N | Mean | SD | N | Mean | SD | N | Mean | SD | N | Mean | SD |
| Buenos Aires | Argentina | 100 | 2.70 | 1.74 | 100 | 2.70 | 1.56 | 100 | 3.05 | 1.29 | 100 | 2.77 | 1.08 |
| Charleroi | Belgium | 82 | 2.10 | 1.29 | 75 | 1.76 | 1.07 | 82 | 2.41 | 0.92 | 75 | 2.25 | 0.85 |
| Ghent | Belgium | 100 | 2.03 | 1.15 | 100 | 2.12 | 1.20 | 100 | 2.24 | 0.78 | 100 | 2.15 | 0.72 |
| Xingu | Brazil | 99 | 0.10 | 0.22 | 99 | 0.08 | 0.20 | 99 | 0.20 | 0.53 | 99 | 0.20 | 0.39 |
| Yanomamo | Brazil | 99 | 0.01 | 0.03 | 96 | 0.01 | 0.02 | 99 | 0.01 | 0.03 | 96 | 0.02 | 0.04 |
| Labrador | Canada | 78 | 3.00 | 1.84 | 83 | 2.49 | 1.98 | 78 | 4.17 | 2.25 | 83 | 3.11 | 1.70 |
| St John's | Canada | 100 | 3.40 | 2.65 | 100 | 3.39 | 1.95 | 100 | 3.62 | 1.71 | 100 | 3.55 | 1.26 |
| Tuquerres | Colombia | 96 | 3.09 | 1.37 | 95 | 2.85 | 1.43 | 96 | 3.06 | 0.91 | 95 | 2.74 | 0.93 |
| Glostrup | Denmark | 99 | 1.88 | 1.29 | 100 | 1.68 | 0.92 | 99 | 2.22 | 0.94 | 100 | 2.19 | 0.81 |
| Bernried | Germany (West Germany) | 99 | 2.14 | 1.04 | 98 | 2.26 | 1.29 | 99 | 2.44 | 0.88 | 98 | 2.46 | 1.04 |
| Heidelberg | Germany (West Germany) | 97 | 1.97 | 0.87 | 99 | 1.79 | 0.93 | 97 | 2.54 | 0.88 | 99 | 2.42 | 0.91 |
| Joensuu | Finland | 100 | 2.22 | 1.28 | 100 | 1.86 | 0.99 | 100 | 2.41 | 0.83 | 100 | 2.24 | 0.81 |
| Turku | Finland | 100 | 1.98 | 1.11 | 100 | 1.67 | 0.76 | 100 | 2.23 | 0.93 | 100 | 2.06 | 0.91 |
| Cottbus | Germany (East Germany) | 99 | 2.78 | 1.46 | 99 | 2.56 | 1.21 | 99 | 2.92 | 1.07 | 99 | 2.55 | 0.70 |
| Porcsalma | Hungary | 100 | 3.82 | 1.71 | 100 | 4.65 | 2.78 | 100 | 4.10 | 1.50 | 100 | 4.18 | 1.49 |
| Reykjavik | Iceland | 100 | 2.07 | 1.13 | 100 | 1.86 | 0.94 | 100 | 2.25 | 0.70 | 100 | 2.20 | 0.83 |
| Ladakh | India | 100 | 3.81 | 2.15 | 100 | 6.04 | 2.84 | 100 | 4.24 | 1.97 | 100 | 5.52 | 2.03 |
| New Delhi | India | 100 | 3.01 | 1.42 | 99 | 3.07 | 1.94 | 100 | 3.65 | 1.23 | 99 | 3.38 | 1.08 |
| Bassiano | Italy | 99 | 3.29 | 1.74 | 100 | 3.40 | 1.67 | 99 | 3.21 | 0.91 | 100 | 3.38 | 1.01 |
| Gubbio | Italy | 99 | 2.83 | 1.35 | 100 | 2.78 | 1.51 | 99 | 3.37 | 1.20 | 100 | 3.21 | 1.07 |
| Mirano | Italy | 100 | 2.71 | 1.48 | 100 | 2.79 | 2.72 | 100 | 3.16 | 1.19 | 100 | 3.08 | 0.87 |
| Naples | Italy | 100 | 2.15 | 0.90 | 100 | 2.21 | 0.94 | 100 | 2.93 | 0.93 | 100 | 2.87 | 0.92 |
| Osaka | Japan | 100 | 3.20 | 1.36 | 97 | 3.24 | 1.53 | 100 | 4.20 | 1.27 | 97 | 3.89 | 1.14 |
| Tochigi | Japan | 95 | 4.61 | 2.36 | 99 | 4.69 | 2.55 | 95 | 4.05 | 1.33 | 99 | 4.24 | 1.29 |
| Toyama | Japan | 100 | 3.89 | 1.63 | 100 | 4.27 | 1.98 | 100 | 4.58 | 1.25 | 100 | 4.64 | 1.51 |
| Rambugu-Ndori | Kenya | 90 | 1.54 | 1.27 | 86 | 1.82 | 1.37 | 90 | 1.94 | 1.04 | 86 | 1.89 | 1.07 |
| Dingli | Malta | 100 | 2.27 | 1.21 | 100 | 2.27 | 1.85 | 100 | 2.48 | 0.84 | 100 | 2.65 | 1.44 |
| Tarahumara | Mexico | 91 | 3.18 | 2.12 | 81 | 3.95 | 2.95 | 91 | 3.27 | 1.37 | 81 | 3.81 | 1.68 |
| Zutphen | The Netherlands | 100 | 2.06 | 1.06 | 99 | 1.77 | 1.21 | 100 | 2.21 | 0.82 | 99 | 2.13 | 0.87 |
| Asaro valley | Papua New Guinea | 88 | 0.50 | 0.58 | 74 | 0.45 | 0.44 | 88 | 0.70 | 0.65 | 74 | 0.55 | 0.45 |
| Beijing | China | 100 | 4.68 | 2.37 | 100 | 4.16 | 2.02 | 100 | 6.35 | 1.81 | 100 | 5.68 | 2.07 |
| Nanning | China | 100 | 5.67 | 2.67 | 100 | 4.98 | 2.64 | 100 | 6.88 | 1.85 | 100 | 5.93 | 2.32 |
| Tianjin | China | 100 | 8.00 | 4.24 | 100 | 7.38 | 4.06 | 100 | 7.67 | 2.25 | 100 | 7.49 | 2.59 |
| Krakow | Poland | 100 | 3.61 | 1.37 | 100 | 3.58 | 1.50 | 100 | 4.14 | 1.16 | 100 | 3.56 | 1.23 |
| Warsaw | Poland | 100 | 3.40 | 1.78 | 100 | 2.92 | 1.42 | 100 | 4.31 | 1.58 | 100 | 3.82 | 1.62 |
| Cartaxo | Portugal | 99 | 2.54 | 1.40 | 99 | 2.40 | 1.40 | 99 | 3.01 | 0.99 | 99 | 2.79 | 1.13 |
| Pusan | South Korea | 100 | 3.93 | 1.93 | 98 | 3.68 | 1.79 | 100 | 4.59 | 1.38 | 98 | 4.13 | 1.44 |
| Moscow | Russia (Soviet Union) | 97 | 2.78 | 1.30 | 97 | 2.91 | 1.49 | 97 | 3.43 | 1.17 | 97 | 3.42 | 1.23 |
| Manresa | Spain | 100 | 2.61 | 1.69 | 100 | 2.29 | 1.28 | 100 | 2.83 | 0.87 | 100 | 2.54 | 0.94 |
| Torrejon | Spain | 100 | 2.59 | 1.33 | 100 | 2.36 | 1.24 | 100 | 2.92 | 1.03 | 100 | 2.68 | 0.86 |
| San Chilo | Taiwan | 89 | 3.72 | 2.00 | 92 | 3.58 | 2.09 | 89 | 4.96 | 2.10 | 92 | 4.82 | 2.18 |
| Plymouth-Bethesda | Trinidad and Tobago | 84 | 2.51 | 1.46 | 92 | 1.82 | 1.03 | 84 | 3.24 | 1.45 | 92 | 2.89 | 1.21 |
| Belfast | United Kingdom | 99 | 2.21 | 1.21 | 100 | 2.40 | 1.34 | 99 | 2.79 | 1.05 | 100 | 2.79 | 1.03 |
| Birmingham | United Kingdom | 100 | 2.18 | 1.10 | 100 | 2.35 | 1.37 | 100 | 2.52 | 0.88 | 100 | 2.67 | 1.06 |
| South Wales | United Kingdom | 100 | 2.68 | 1.61 | 99 | 2.34 | 1.37 | 100 | 2.57 | 0.87 | 99 | 2.51 | 0.85 |
| Chicago | United States | 97 | 2.25 | 1.21 | 99 | 2.46 | 1.84 | 97 | 2.78 | 1.23 | 99 | 2.83 | 1.33 |
| Goodman - Black | United States | 93 | 3.99 | 2.87 | 93 | 3.50 | 2.11 | 93 | 4.68 | 2.63 | 93 | 4.90 | 2.75 |
| Goodman - White | United States | 99 | 3.08 | 1.93 | 99 | 2.77 | 1.68 | 99 | 3.22 | 1.32 | 99 | 3.10 | 1.13 |
| Hawaii | United States | 94 | 3.19 | 1.99 | 93 | 3.20 | 1.81 | 94 | 3.43 | 1.62 | 93 | 3.64 | 2.04 |
| Jackson - Black | United States | 84 | 3.39 | 1.77 | 100 | 3.32 | 2.17 | 84 | 4.05 | 2.42 | 100 | 4.09 | 1.87 |
| Jackson - White | United States | 100 | 2.33 | 1.36 | 99 | 2.10 | 1.30 | 100 | 2.69 | 1.24 | 99 | 2.86 | 1.45 |
| Harare | Zimbabwe | 100 | 3.43 | 2.22 | 95 | 3.35 | 2.14 | 100 | 4.02 | 2.21 | 95 | 4.01 | 1.89 |

Na, sodium; K, potassium; SD, standard deviation.

Data for sodium, potassium, Na/K ratio and urine volume are previously published by gender and age in The INTERSALT Co-operative Research Group. Appendix tables. Centre-specific results by age and sex. *J Hum Hypertens*. 1989 Oct;3(5):331-407.

**Supplementary Table 3. Proportion of study participants in specific ethnic groups by population.**

| **Sample** | | N | White | Black | Native American | Asian-Indian | East Asian | Others |
| --- | --- | --- | --- | --- | --- | --- | --- | --- |
| Buenos Aires | Argentina | 200 | 91.5% | 0.0% | 0.5% | 0.0% | 0.0% | 8.0% |
| Charleroi | Belgium | 157 | 100.0% | 0.0% | 0.0% | 0.0% | 0.0% | 0.0% |
| Ghent | Belgium | 200 | 99.0% | 0.5% | 0.0% | 0.0% | 0.0% | 0.5% |
| Xingu | Brazil | 198 | 1.0% | 0.0% | 99.0% | 0.0% | 0.0% | 0.0% |
| Yanomamo | Brazil | 195 | 0.0% | 0.0% | 100.0% | 0.0% | 0.0% | 0.0% |
| Labrador | Canada | 161 | 24.2% | 0.0% | 0.0% | 0.0% | 0.0% | 75.8% |
| St John's | Canada | 200 | 100.0% | 0.0% | 0.0% | 0.0% | 0.0% | 0.0% |
| Tuquerres | Colombia | 191 | 8.9% | 0.0% | 0.0% | 0.0% | 0.0% | 91.1% |
| Glostrup | Denmark | 199 | 98.5% | 0.0% | 0.0% | 0.5% | 0.0% | 1.0% |
| Bernried | Germany (West Germany) | 197 | 100.0% | 0.0% | 0.0% | 0.0% | 0.0% | 0.0% |
| Heidelberg | Germany (West Germany) | 196 | 100.0% | 0.0% | 0.0% | 0.0% | 0.0% | 0.0% |
| Joensuu | Finland | 200 | 100.0% | 0.0% | 0.0% | 0.0% | 0.0% | 0.0% |
| Turku | Finland | 200 | 100.0% | 0.0% | 0.0% | 0.0% | 0.0% | 0.0% |
| Cottbus | Germany (East Germany) | 198 | 100.0% | 0.0% | 0.0% | 0.0% | 0.0% | 0.0% |
| Porcsalma | Hungary | 200 | 94.5% | 0.0% | 0.0% | 0.0% | 0.0% | 5.5% |
| Reykjavik | Iceland | 200 | 100.0% | 0.0% | 0.0% | 0.0% | 0.0% | 0.0% |
| Ladakh | India | 200 | 0.0% | 0.0% | 0.0% | 100.0% | 0.0% | 0.0% |
| New Delhi | India | 199 | 0.0% | 0.5% | 0.0% | 99.5% | 0.0% | 0.0% |
| Bassiano | Italy | 199 | 100.0% | 0.0% | 0.0% | 0.0% | 0.0% | 0.0% |
| Gubbio | Italy | 199 | 100.0% | 0.0% | 0.0% | 0.0% | 0.0% | 0.0% |
| Mirano | Italy | 200 | 100.0% | 0.0% | 0.0% | 0.0% | 0.0% | 0.0% |
| Naples | Italy | 200 | 100.0% | 0.0% | 0.0% | 0.0% | 0.0% | 0.0% |
| Osaka | Japan | 197 | 0.0% | 0.0% | 0.0% | 0.0% | 100.0% | 0.0% |
| Tochigi | Japan | 194 | 0.0% | 0.0% | 0.0% | 0.0% | 100.0% | 0.0% |
| Toyama | Japan | 200 | 0.0% | 0.0% | 0.0% | 0.0% | 100.0% | 0.0% |
| Rambugu-Ndori | Kenya | 176 | 0.0% | 100.0% | 0.0% | 0.0% | 0.0% | 0.0% |
| Dingli | Malta | 200 | 100.0% | 0.0% | 0.0% | 0.0% | 0.0% | 0.0% |
| Tarahumara | Mexico | 172 | 0.0% | 0.0% | 98.3% | 0.0% | 0.0% | 1.7% |
| Zutphen | The Netherlands | 199 | 92.5% | 0.0% | 0.0% | 1.5% | 1.0% | 5.0% |
| Asaro valley | Papua New Guinea | 162 | 0.0% | 0.0% | 0.0% | 0.0% | 0.0% | 100.0% |
| Beijing | China | 200 | 0.0% | 0.0% | 0.0% | 0.0% | 100.0% | 0.0% |
| Nanning | China | 200 | 0.0% | 0.0% | 0.0% | 0.0% | 100.0% | 0.0% |
| Tianjin | China | 200 | 0.0% | 0.0% | 0.0% | 0.0% | 100.0% | 0.0% |
| Krakow | Poland | 200 | 100.0% | 0.0% | 0.0% | 0.0% | 0.0% | 0.0% |
| Warsaw | Poland | 200 | 100.0% | 0.0% | 0.0% | 0.0% | 0.0% | 0.0% |
| Cartaxo | Portugal | 198 | 99.5% | 0.0% | 0.0% | 0.5% | 0.0% | 0.0% |
| Pusan | South Korea | 198 | 0.0% | 0.0% | 0.0% | 0.0% | 100.0% | 0.0% |
| Moscow | Russia (Soviet Union) | 194 | 100.0% | 0.0% | 0.0% | 0.0% | 0.0% | 0.0% |
| Manresa | Spain | 200 | 99.5% | 0.0% | 0.0% | 0.0% | 0.0% | 0.5% |
| Torrejon | Spain | 200 | 100.0% | 0.0% | 0.0% | 0.0% | 0.0% | 0.0% |
| San Chilo | Taiwan | 181 | 0.0% | 0.0% | 0.0% | 0.0% | 100.0% | 0.0% |
| Plymouth-Bethesda | Trinidad and Tobago | 176 | 0.0% | 98.3% | 0.0% | 0.0% | 0.6% | 1.1% |
| Belfast | United Kingdom | 199 | 100.0% | 0.0% | 0.0% | 0.0% | 0.0% | 0.0% |
| Birmingham | United Kingdom | 200 | 95.0% | 3.5% | 0.0% | 1.5% | 0.0% | 0.0% |
| South Wales | United Kingdom | 199 | 99.5% | 0.0% | 0.0% | 0.0% | 0.5% | 0.0% |
| Chicago | United States | 196 | 67.9% | 30.1% | 0.0% | 0.0% | 2.0% | 0.0% |
| Goodman - Black | United States | 186 | 0.0% | 100.0% | 0.0% | 0.0% | 0.0% | 0.0% |
| Goodman - White | United States | 198 | 99.5% | 0.5% | 0.0% | 0.0% | 0.0% | 0.0% |
| Hawaii | United States | 187 | 0.0% | 0.0% | 0.0% | 0.0% | 0.0% | 100.0% |
| Jackson - Black | United States | 184 | 0.0% | 100.0% | 0.0% | 0.0% | 0.0% | 0.0% |
| Jackson - White | United States | 199 | 100.0% | 0.0% | 0.0% | 0.0% | 0.0% | 0.0% |
| Harare | Zimbabwe | 195 | 0.0% | 98.5% | 0.0% | 0.0% | 0.0% | 1.5% |

**Supplementary Table 4. Regression coefficients of 24-hour urine Na/K ratio on casual urine Na/K ratio by individual level linear regression analysis.**

| Population sample | Regression coefficients | SE | P values for interaction |
| --- | --- | --- | --- |
| Individuals ages 20-24 (N=1,137) | 0.613 | 0.017 | 0.118 |
| Individuals ages 25-34 (N=2,662) | 0.598 | 0.012 |
| Individuals ages 35-44 (N=2,533) | 0.608 | 0.013 |
| Individuals ages 45-54 (N=2,564) | 0.613 | 0.013 |
| Individuals ages 55-59 (N=1,169) | 0.662 | 0.020 |
| Individual men (N=5,039) | 0.632 | 0.009 | 0.0039 |
| Individual women (N=5,026) | 0.595 | 0.009 |
| White individuals (N=5,853) | 0.444 | 0.011 | <0.0001 |
| Black individuals (N=980) | 0.485 | 0.019 |
| Native American individuals (N=561) | 0.681 | 0.025 |
| Asian-Indian individuals (N=406) | 0.522 | 0.025 |
| East Asian individuals (N=1,578) | 0.498 | 0.011 |
| Individuals of other ethnicities (Others) (N=687) | 0.703 | 0.025 |
| Individuals taking neither anti-hypertensive medications nor potassium (N=7,629) | 0.638 | 0.007 | <0.0001 |
| Individuals taking anti-hypertensive medications or potassium (N=2,436) | 0.513 | 0.014 |

Anti-hypertensive medications include potassium sparing diuretics, other diuretics, and other anti-hypertensive drugs affecting BP.

East Asian individuals are defined as Chinese, Japanese and Korean individuals. Details of the definition of ethnic groups are defined in reference 34.

**Supplementary Figure 1**

Plots of Na/K ratio of casual urine versus 24-hour urine, and Bland-Altman plots by sex (52 population samples).

Pearson correlation coefficients between 24-hour urinary Na/K ratio and casual urinary sodium/potassium ratio were r=0.95 to 0.96 in sex specific analyses for 52 populations. The bias values between 24-hour urinary sodium/potassium ratio and casual urinary sodium/potassium by Bland-Altman method were 0.42 and 0.36 in men and women, respectively. Others are nine population samples from Argentina, Colombia, Mexico, Trinidad and Tobago, Zimbabwe, Yanomamo and Xingu Indians in Brazil, Kenya and Papua New Guinea; these 9 samples were neither western populations (N=33) nor Asian populations (N=10).

**Supplementary Figure 2**

Plots of Na/K ratio of casual urine versus 24-hour urine, and Bland-Altman plots by age (52 population samples).

Pearson correlation coefficients between 24-hour urinary sodium/potassium ratio and casual urinary sodium/potassium ratio were r=0.94 to 0.95 in age specific analyses for 52 population samples. The bias values between 24-hour urinary sodium/potassium ratio and casual urinary sodium/potassium by Bland-Altman method were 0.32 to 0.46. Others are nine population samples from Argentina, Colombia, Mexico, Trinidad and Tobago, Zimbabwe, Yanomamo and Xingu Indians in Brazil, Kenya and Papua New Guinea; these 9 samples were neither western populations (N=33) nor Asian populations (N=10).

**Supplementary Figure 3**

Plots of Na/K ratio of casual urine versus 24-hour urine, and Bland-Altman plots, Western and Asian population samples.

Pearson correlation coefficients between 24-hour urinary sodium/potassium ratio and casual urinary sodium/potassium ratio were r=0.92 and 0.88 in analyses for Western and Asian populations, respectively. The bias values between 24-hour urinary sodium/potassium ratio and casual urinary sodium/potassium by Bland-Altman method were 0.39 to 0.52.

**Supplementary Figure 4**

Plots of Na/K ratio of casual urine versus 24-hour urine, and Bland-Altman plots by sex (10,065 individuals).

Pearson correlation coefficients between 24-hour urinary sodium/potassium ratio and casual urinary sodium/potassium ratio were r=0.68 to 0.70 in sex specific analyses for individuals. The bias values between 24-hour urinary sodium/potassium ratio and casual urinary sodium/potassium by Bland-Altman method were 0.37 to 0.42.

**Supplementary Figure 5**

Plots of Na/K ratio of casual urine versus 24-hour urine, and Bland-Altman plots by ethnic group (10,065 individuals).

Pearson correlation coefficients between 24-hour urinary sodium/potassium ratio and casual urinary sodium/potassium ratio were r=0.47 to 0.81 in analyses for various ethnic groups. The bias values between 24-hour urinary sodium/potassium ratio and casual urinary sodium/potassium by Bland-Altman method were 0.04 to 0.69. East Asian populations are defined as eight population samples from Japan, China, South Korea and Taiwan.

**Supplementary Figure 6**

Plots of Na/K ratio of casual urine versus 24-hour urine, and Bland-Altman plots by age (10,065 individuals).

Pearson correlation coefficients between 24-hour urinary sodium/potassium ratio and casual urinary sodium/potassium ratio were r=0.66 to 0.72 from analyses in age-specific individuals. The bias values between 24-hour urinary sodium/potassium ratio and casual urinary sodium/potassium by Bland-Altman method were 0.33 to 0.45.

**Supplementary Figure 7**

Plots of Na/K ratio of casual urine versus 24-hour urine, and Bland-Altman plots by anti-hypertensive medication use (10,065 individuals).

Pearson correlation coefficients between 24-hour urinary sodium/potassium ratio and casual urinary sodium/potassium ratio were r=0.58 and 0.72 in analyses of subgroups on anti-hypertensive medication and potassium supplement intake, respectively. The bias values between 24-hour urinary sodium/potassium ratio and casual urinary sodium/potassium by Bland-Altman method were 0.38 and 0.45.

**Supplementary Figure 1.**


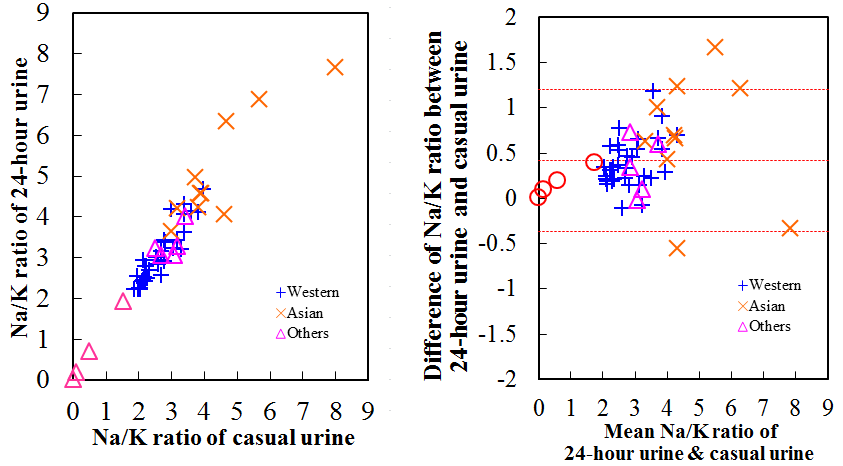

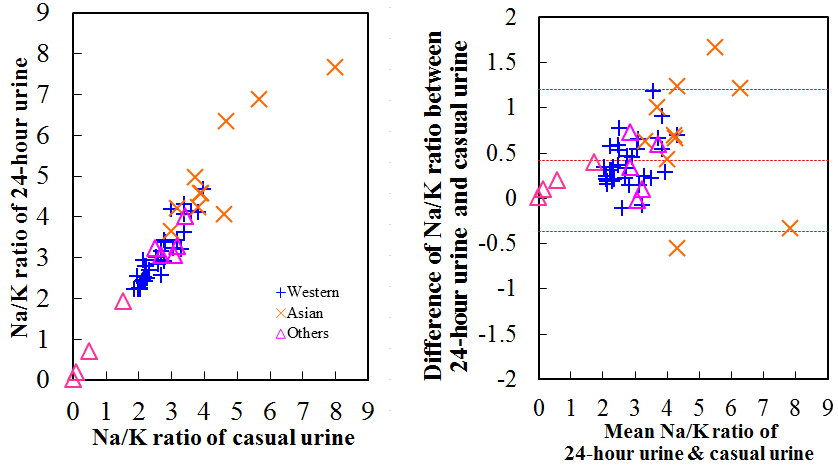


**r=0.96**

**Upper limit 1.20**

**Bias 0.42**

**Lower limit -0.37**

(a) Men (52 populations)


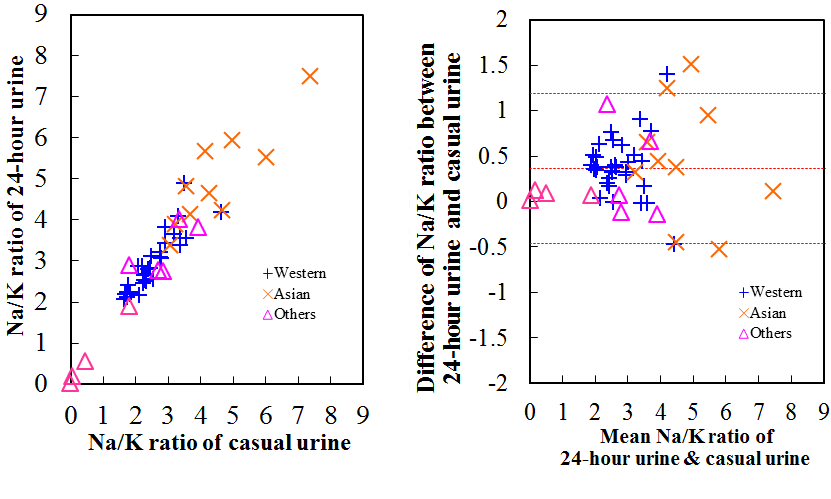


**r=0.95**

(b) Women (52 populations)

**Upper limit 1.18**

**Lower limit -0.46**

**Bias 0.36**

**Supplementary Figure 2.**


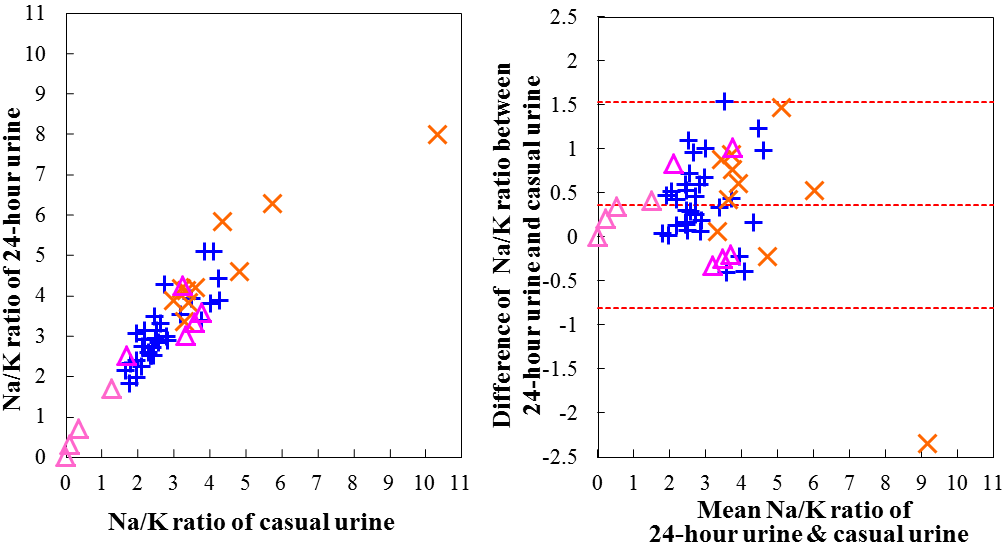


(a) Age 20-24 (52 populations)

**r=0.92**

**Upper limit 1.54**

**Lower limit -0.81**

**Bias 0.36**


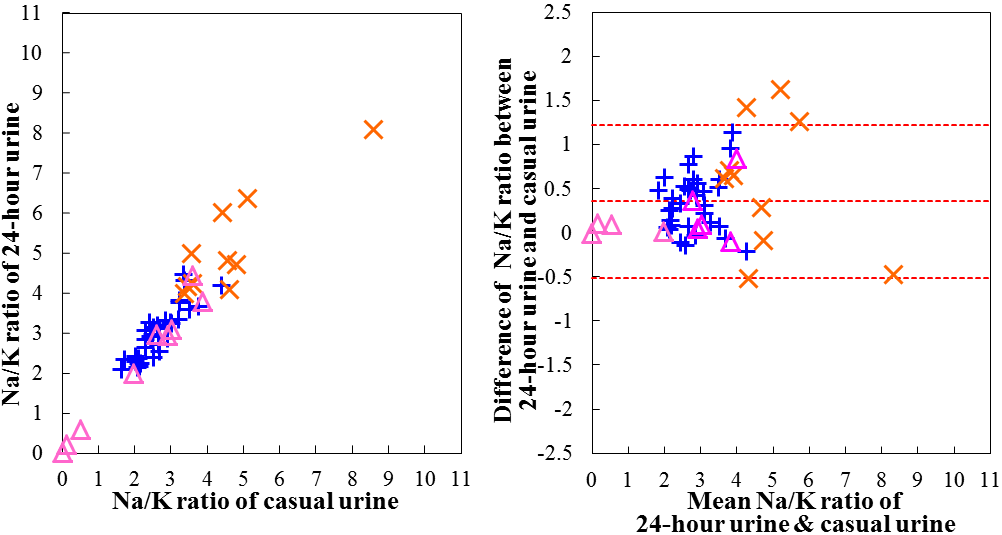


**r=0.95**

(b) Age 25-34 (52 populations)

**Upper limit 1.22**

**Lower limit -0.51**

**Bias 0.36**


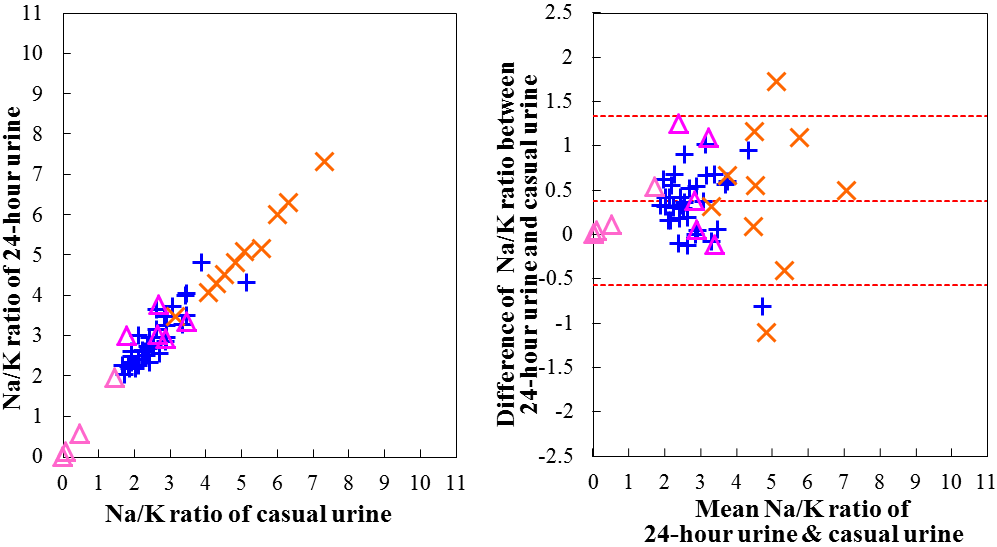


**r=0.93**

**Upper limit 1.33**

**Lower limit -0.58**

**Bias 0.38**

(c) Age 35-44 (52 populations)


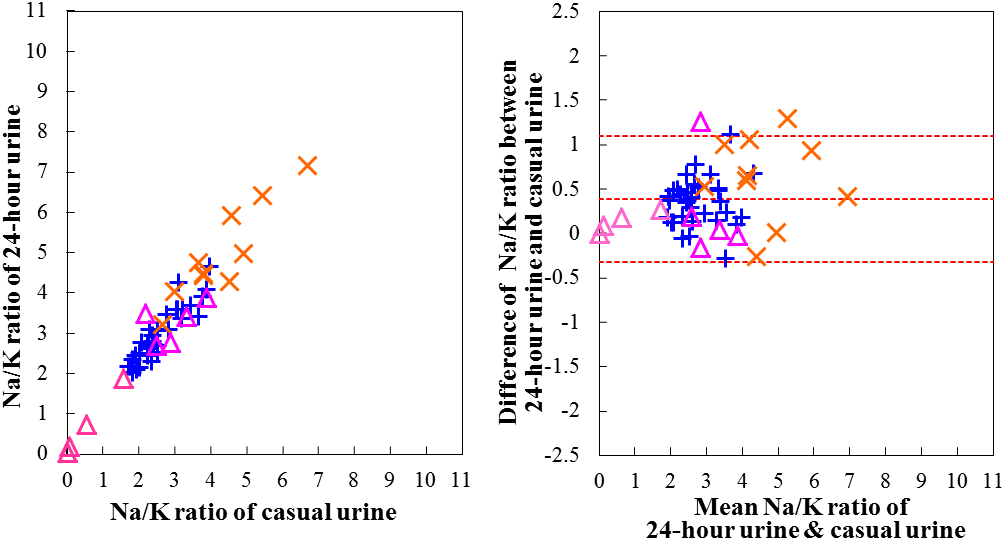


**r=0.96**

(d) Age 45-54 (52 populations)

**Upper limit 1.09**

**Lower limit -0.33**

**Bias 0.38**


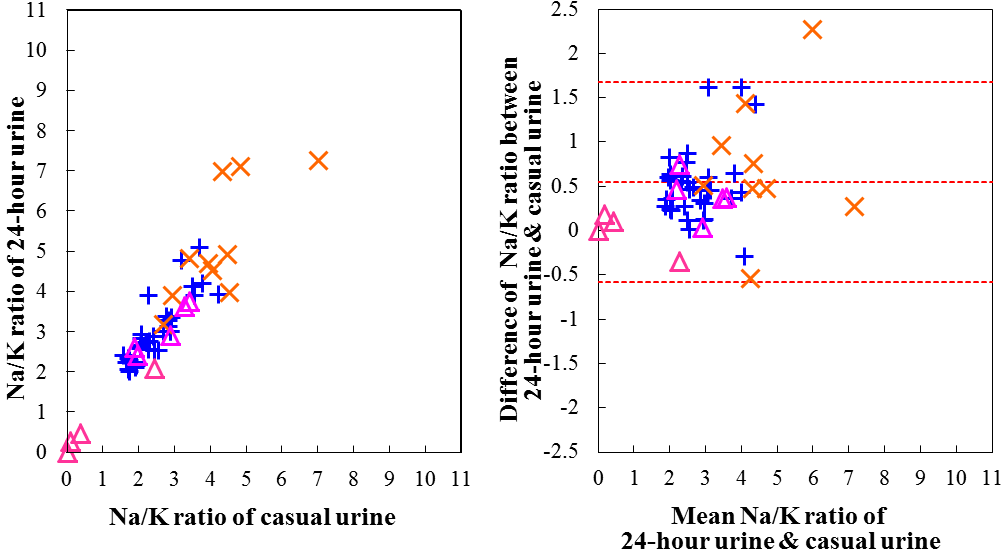


**r=0.92**

**Upper limit 1.67**

**Lower limit -0.59**

**Bias 0.54**

(e) Age 55-59 (52 populations)

**Supplementary Figure 3.**


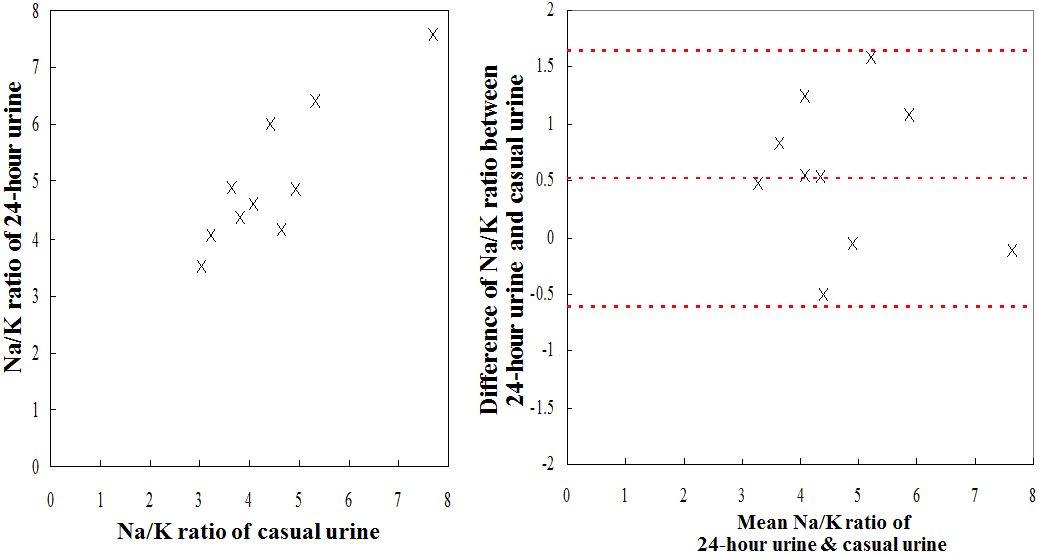


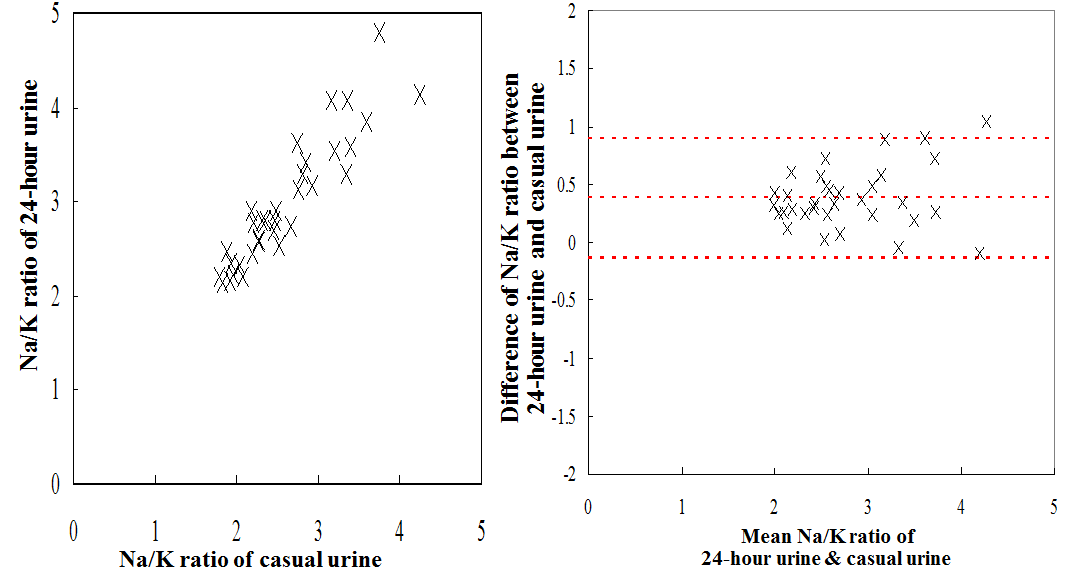


**r=0.92**

(a) Western populations (33 populations)

**Upper limit 0.90**

**Lower limit -0.13**

**Bias 0.39**

**r=0.88**

(b) Asian populations (10 populations)

**Upper limit 1.65**

**Lower limit -0.60**

**Bias 0.52**

**Supplementary Figure 4.**


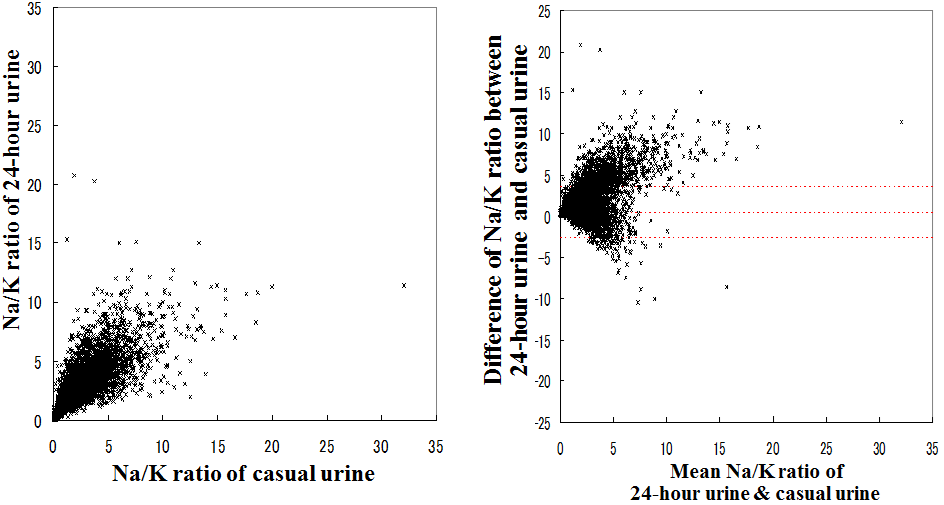


**r=0.70**

**Upper limit 3.53**

**Lower limit -2.60**

**Bias 0.42**

(a) Individual men (n=5,039)


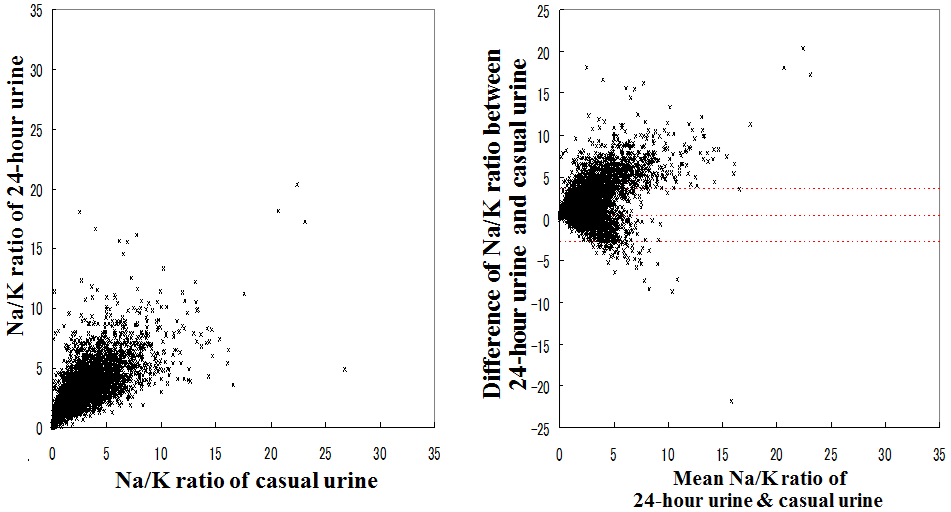


**r=0.68**

**Upper limit 3.58**

**Bias 0.37**

**Lower limit -2.84**

(b) Individual women (n=5,026)

**Supplementary Figure 5.**


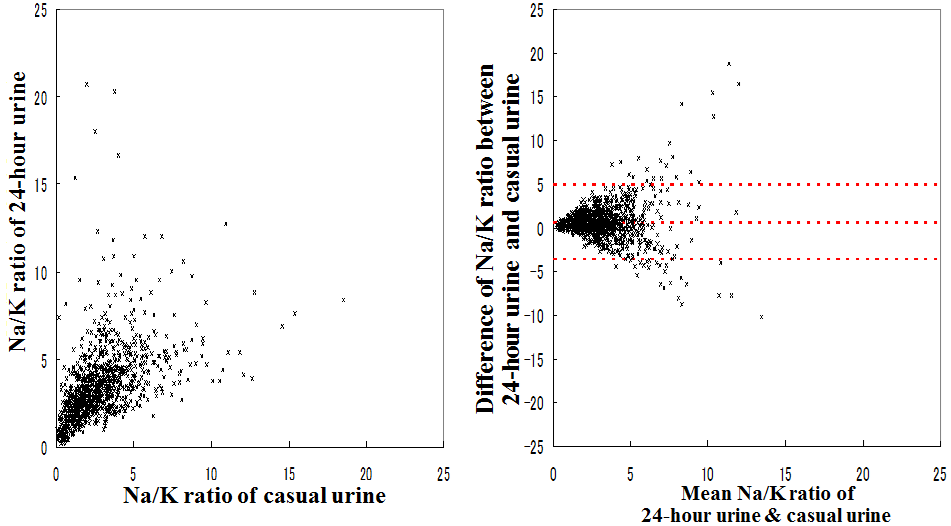


**r=0.47**

**Upper limit 4.97**

**Lower limit -3.60**

**Bias 0.69**

(b) Black (n=980)


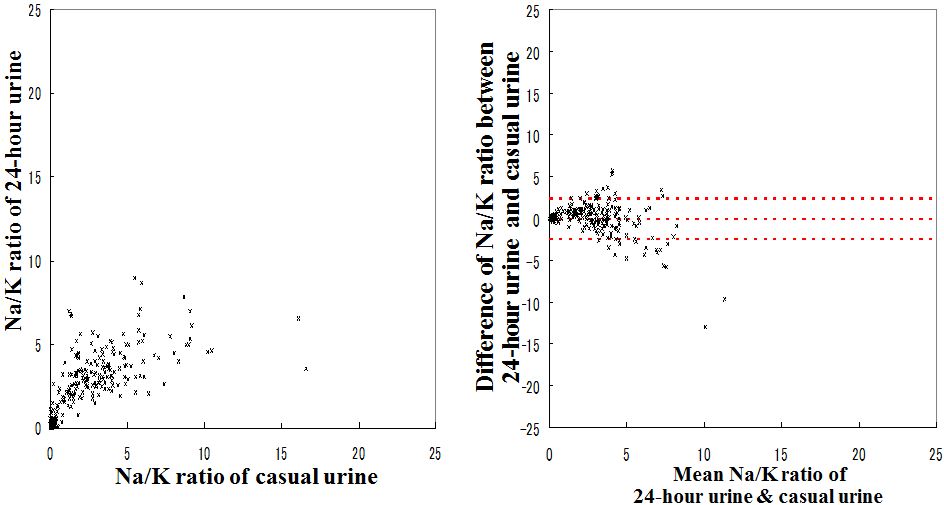


**r=0.81**

**Upper limit 2.49**

**Lower limit -2.42**

**Bias 0.04**

(c) Native American (n=561)


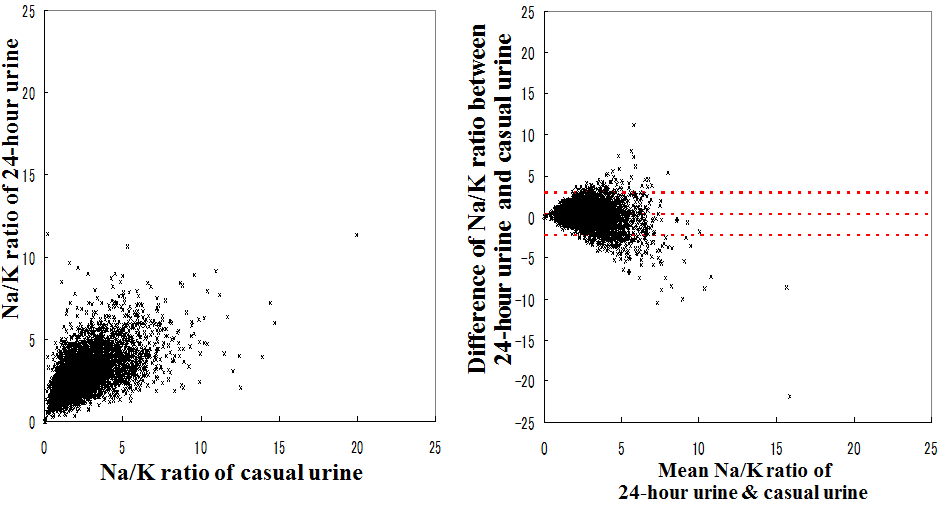


**r=0.58**

(a) White (n=5,853)

**Upper limit 2.91**

**Lower limit -2.24**

**Bias 0.33**


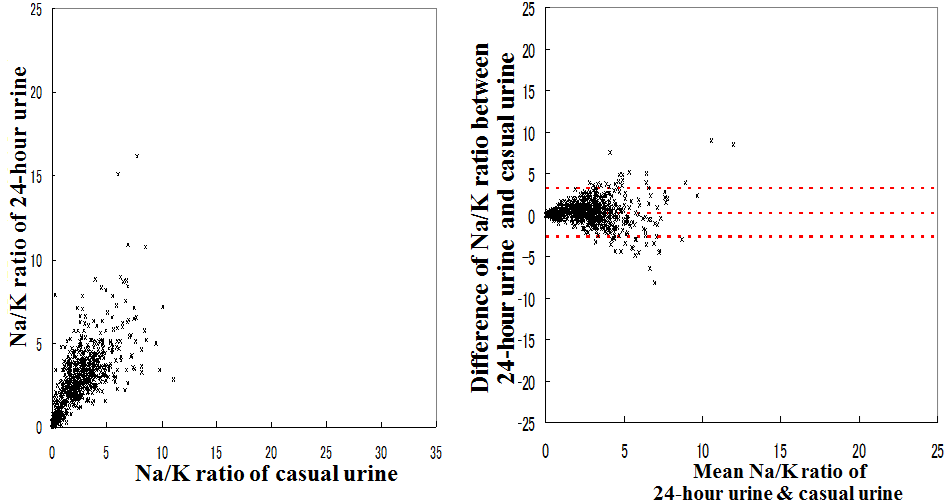

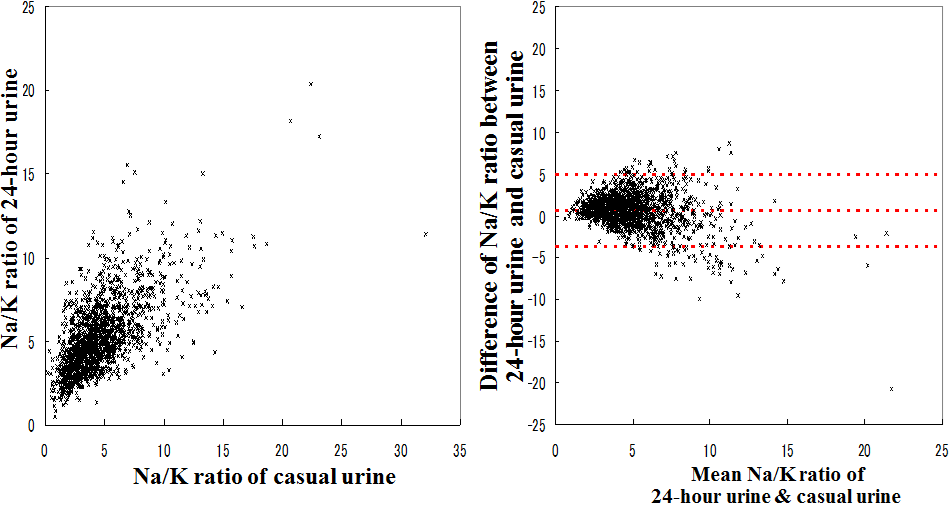

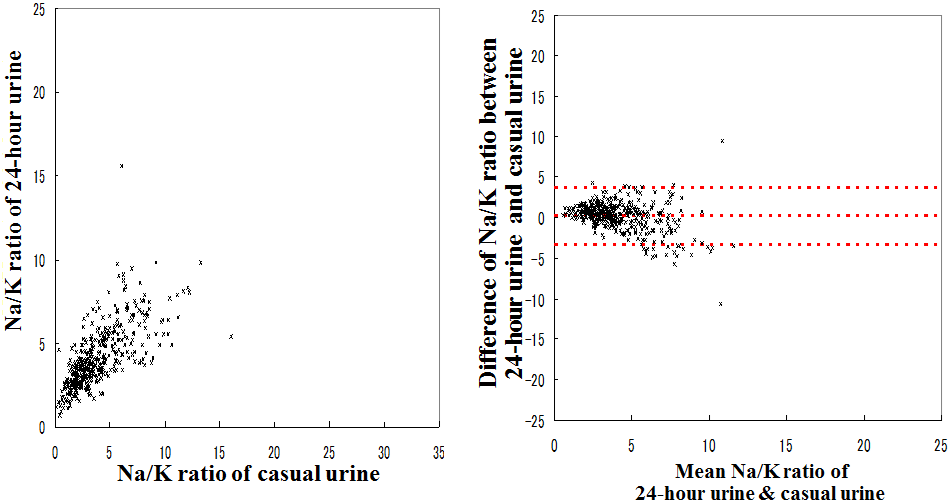


**Upper limit 4.90**

**Bias 0.65**

**Lower limit -3.61**

**r=0.70**

**r=0.64**

**r=0.70**

(d) Asian-Indian (n=406)

**Upper limit 3.67**

**Lower limit -3.23**

**Bias 0.22**

(e) East Asian (n=1,578)

(f) Others (n=687)

**Upper limit 3.24**

**Lower limit -2.61**

**Bias 0.31**

**Supplementary Figure 6.**


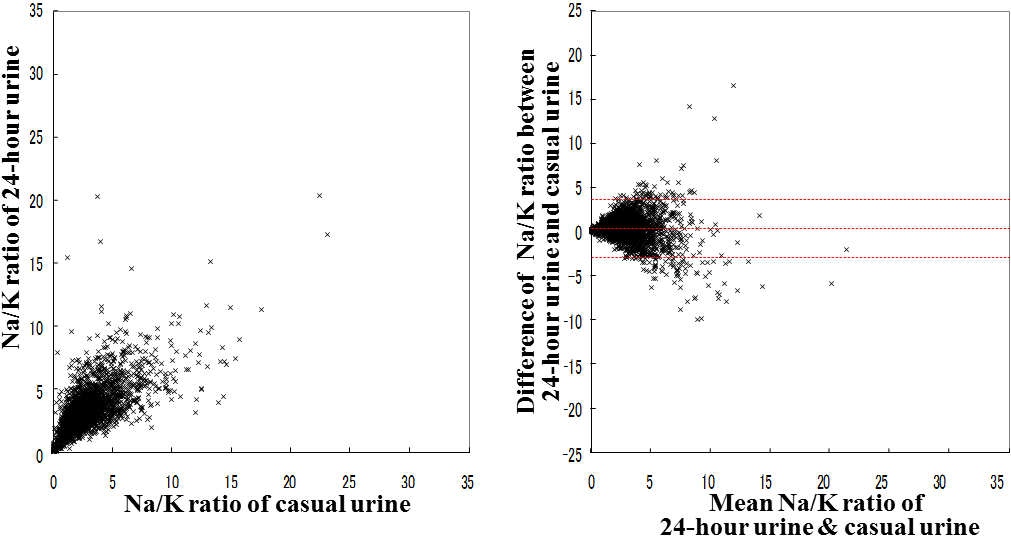

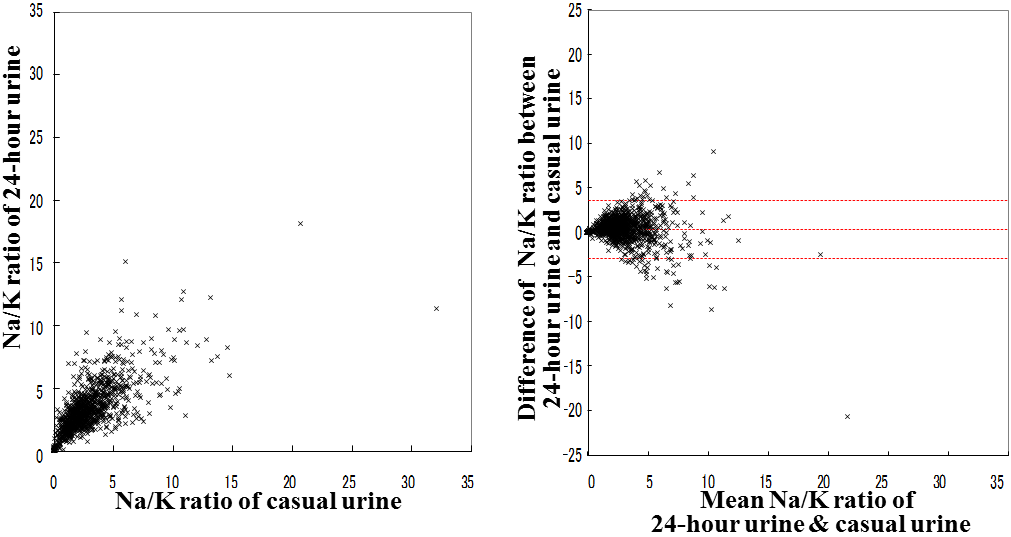

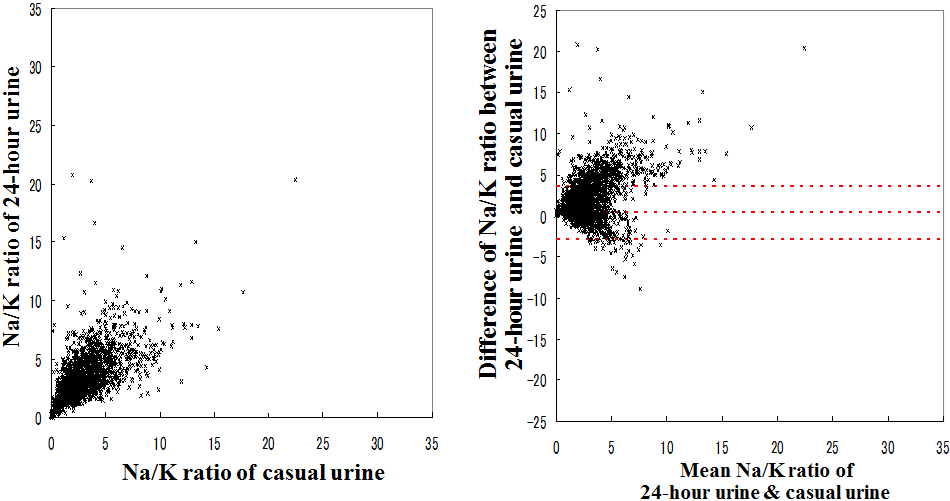

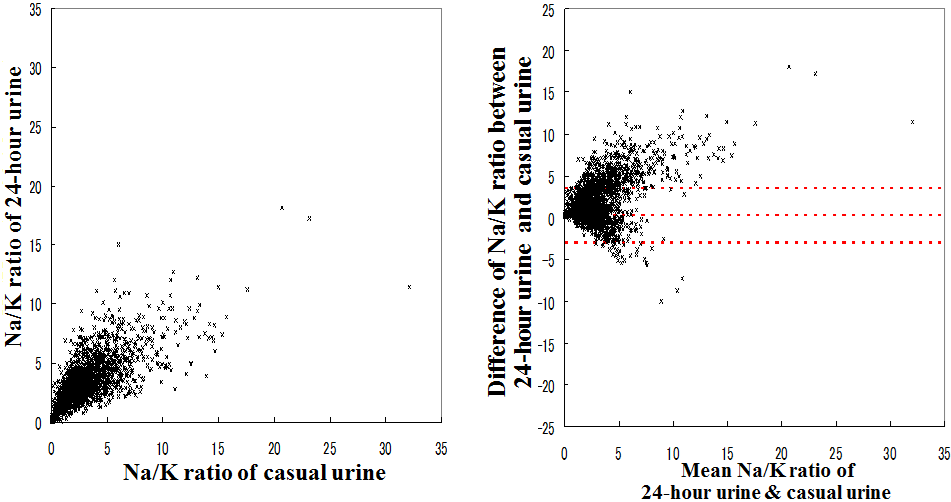


(a) Age 20-24 (n=1,137)

(b) Age 25-34 (n=2,662)

**r=0.72**

**r=0.68**

**Upper limit 3.68**

**Lower limit -2.93**

**Bias 0.37**

**Upper limit 3.64**

**Lower limit -2.91**

**Bias 0.37**


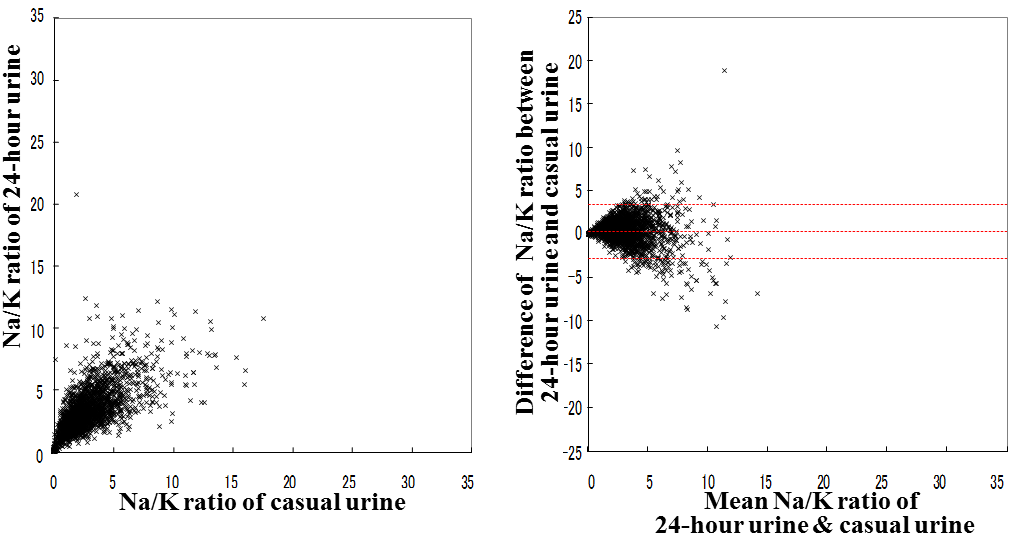

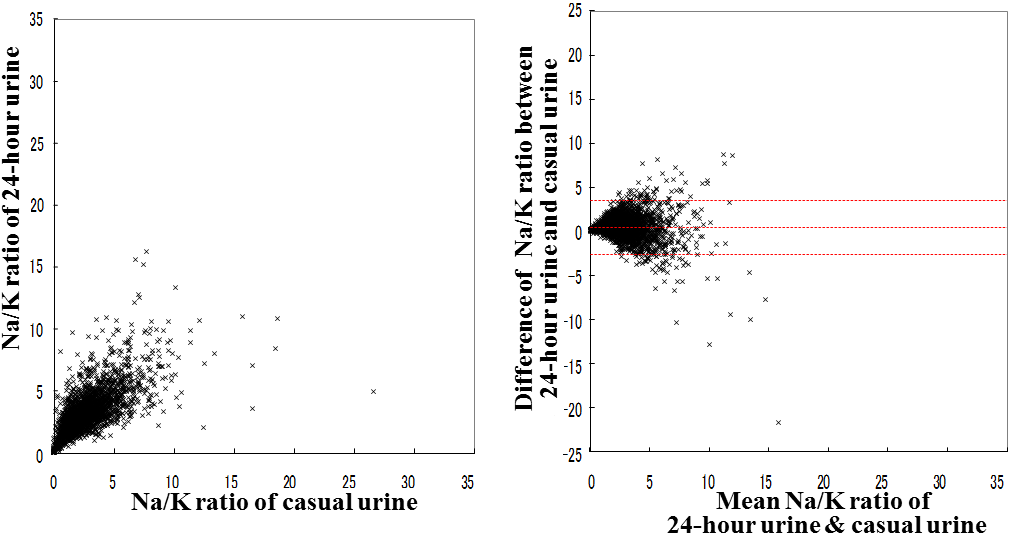


(d) Age 45-54 (n=2,564)

(c) Age 35-44 (n=2,533)

**r=0.69**

**r=0.69**

**Upper limit 3.45**

**Lower limit -2.65**

**Bias 0.40**

**Upper limit 3.49**

**Lower limit -2.73**

**Bias 0.38**


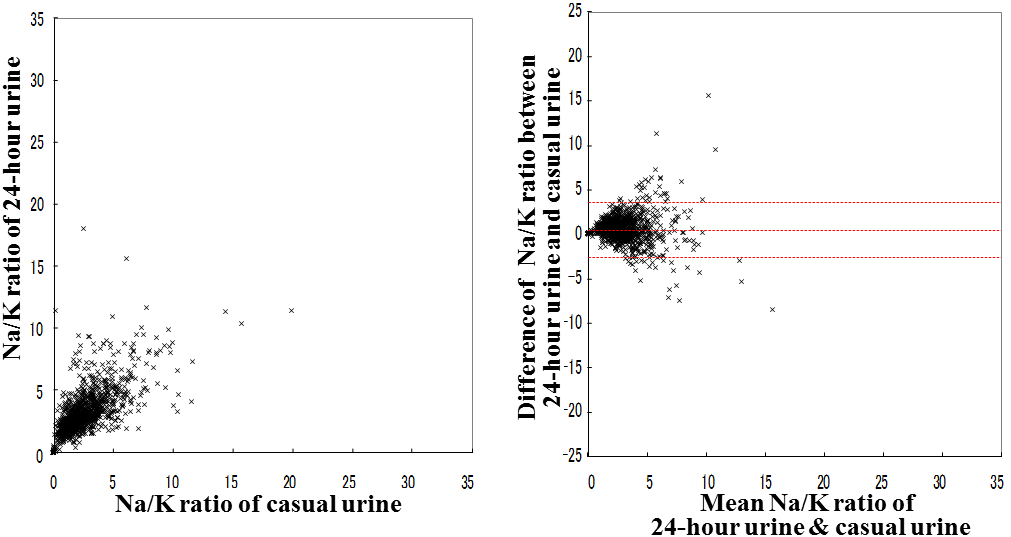


(e) Age 55-59 (n=1,169)

**r=0.68**

**Upper limit 3.60**

**Lower limit -2.57**

**Bias 0.51**

**
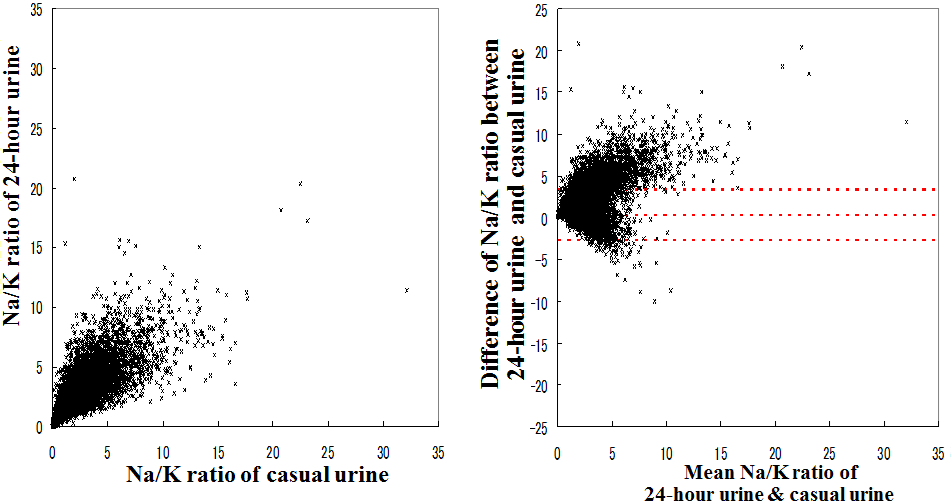

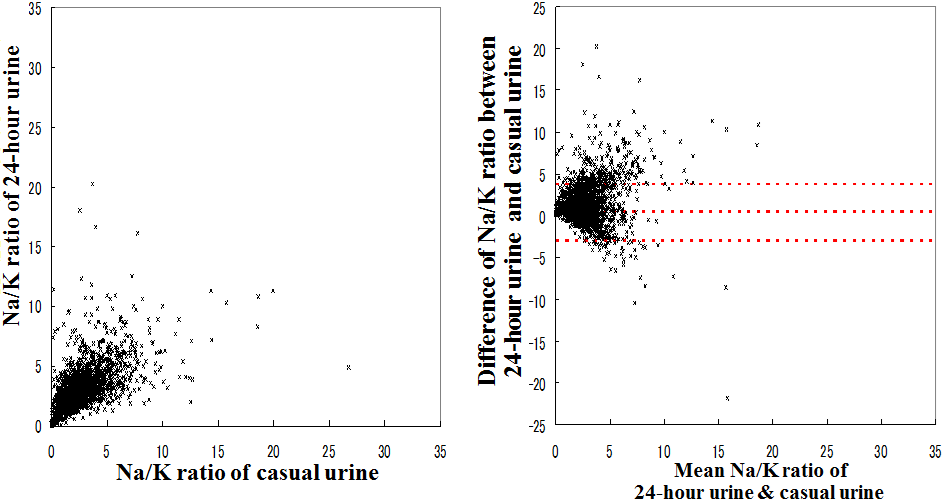
Supplementary Figure 7**

(b) Individuals neither taking anti-hypertensive medications nor potassium supplements (n=7,629)

(a) Individuals taking anti-hypertensive medications or potassium supplements (n=2,436)

**r=0.72**

**Upper limit 3.46**

**Lower limit -2.70**

**Bias 0.38**

**r=0.58**

**Lower limit -2.95**

**Bias 0.45**

**Upper limit 3.40**
